# Global analysis of infant gut microbiota revealed distinctive maturation dynamics across lifestyles

**DOI:** 10.1038/s41522-026-01130-4

**Published:** 2026-08-19

**Authors:** Robert Bücking, Greta Pasquali, Ulrike Löber, Claudia Buss, Dorothee Viemann, Víctor Hugo Jarquín-Díaz, Sofia Kirke Forslund-Startceva

**Affiliations:** 1 Max-Delbrück Center for Molecular Medicine in the Helmholtz Association (MDC), Berlin, Germany; 2 Charité–Universitätsmedizin Berlin, a corporate member of Freie Universität Berlin and Humboldt-Universität zu Berlin, Berlin, Germany; 3 Experimental and Clinical Research Center (ECRC), a cooperation of the Max-Delbrück Center and Charité–Universitätsmedizin, Berlin, Germany; 4 Translational Pediatrics, Department of Pediatrics, University Hospital Würzburg, Würzburg, Germany; 5 Department of Medicine DIMED, University of Padua, Padua, Italy; 6 Institute of Medical Psychology, Charité–Universitätsmedizin Berlin, Corporate Member of Freie Universität Berlin, Humboldt-Universität zu Berlin, and Berlin Institute of Health (BIH), Berlin, Germany; 7 Development, Health and Disease Research Program, University of California, Irvine, Irvine, CA USA; 8 German Center for Child and Adolescent Health (DZKJ), partner site Berlin, Charité - Universitätsmedizin Berlin, Berlin, Germany; 9 German Center for Mental Health (DZPG), partner site Berlin, Charité - Universitätsmedizin Berlin, Berlin, Germany; 10 Center for Infection Research, University Würzburg, Würzburg, Germany; 11 Cluster of Excellence RESIST (EXC 2155), Hannover Medical School, Hannover, Germany; 12 German Center for Cardiovascular Research (DZHK), partner site Berlin, Berlin, Germany; 13 Structural and Computational Biology Unit (SCB), EMBL Heidelberg, Heidelberg, Germany

**Keywords:** Computational biology and bioinformatics, Microbiology

## Abstract

The infant gut microbiome develops during the first years of life and influences long-term health through its interaction with immune system development. However, our understanding of early-life microbiome assembly is biased by the predominance of infants with industrialized lifestyles from North America and Europe. Here, we address this bias by assembling a globally representative dataset of 14,979 infant gut microbiomes from 2746 individuals to train a microbiome maturation model that can characterize lifestyle-specific patterns of microbial maturation as a function of age. Models trained exclusively on industrialized infants perform poorly when applied to non-industrialized datasets. In contrast, more diverse models, including individuals from both lifestyles, achieve increased correlation between microbial and chronological age (Δ*R*^2^ = 0.123). We identified differences in relevant taxa associated with the maturation in the different lifestyles. Additionally, our modeling approach detects a delay in the microbial maturation of independent cohorts of severely malnourished and preterm infants compared to healthy ones. Our results underscore the relevance of global diversity in microbiome research and provide deeper insights into context-dependent maturation dynamics of the infant gut microbiome.

## Introduction

Early-life maturation of the gut microbiome is a key determinant of human health, influencing immune system development, metabolic adjustments, and susceptibility to diseases throughout life^1–3^. However, our understanding of this process is primarily based on infants in industrialized populations, which represent only a small fraction of global diversity^4^. Infants living in non-industrialized or traditional communities remain largely underrepresented in microbiome research, leaving a major gap in understanding how different environments and living conditions shape early-life microbial community assembly.

Immediately after birth, the infant’s gut is rapidly colonized by a variety of bacteria, and it progressively develops until it resembles the adult microbiome by around 3 years of age^5^. The microbiome maturation is influenced by the simultaneous development of the immune system. However, other factors, including delivery mode, gestational age, dietary shifts^6,7^, exposure to antibiotics^8^, and lifestyle^9^ continue to shape the microbiome during all stages of life. Models of microbiome maturation have shown that deviations in the early microbiome development are associated with malnutrition^10^ and health outcomes later in life, such as asthma and allergies^11^, and provide a powerful tool to detect generalizable microbial patterns across cohorts^12^. Thus, the maturation of the early-life microbiome is a compelling model system for investigating ecological succession and health-related microbial dynamics.

Despite the extensive research on the early-life microbiome, most studies remain disproportionately focused on populations from North America and Europe, resulting in a significant geographical bias^4^. Consequently, the diversity in host genetics, ethnicity, and particularly lifestyles is limited in most of the studies, although those factors are known to significantly impact the adult human microbiome^5,9,13–15^. The industrialized lifestyle in those geographical regions involves increased hygiene and exposure to antibiotics, reduced contact with wildlife, and a dietary shift toward more processed, high-caloric foods. Collectively, these factors lead to a reduced microbial diversity and altered community structures in the adult human microbiome^16–21^. Thus, the focus on industrialized populations skews our understanding of global microbiome dynamics, particularly during the critical stages of microbiome maturation in infancy.

To address the gap driven by single cohorts and homogeneous populations in microbiome maturation studies, we performed a globally representative analysis of infant gut microbiome maturation across diverse populations and lifestyles. Our study integrates and analyzes previously publicly available datasets using machine learning models. We specifically compare the microbiome maturation trajectories between infants from industrialized and various non-industrialized populations, comprising a collection of different lifestyles. Thereby, we contribute to a broader understanding of early-life microbial colonization and its implications for human health.

## Results

### Age and lifestyle drive the maturation of the gut microbiome

To investigate the development of the human infant gut microbiome across different lifestyles, we analyzed 11,106 samples from infants with an industrialized lifestyle and 3873 samples from infants with non-industrialized lifestyles from 20 publicly available studies (Table 1, Supplementary Table S1). We detected 890 genera, of which only 61 were present in all studies regardless of the lifestyle. Among the remaining genera, 506 genera were detected in microbiomes from both lifestyles, while 365 genera were exclusive to industrialized microbiomes and 19 were found only in non-industrialized microbiomes (Supplementary Fig. S1A). Of the lifestyle-specific taxa, 97% had a prevalence below 1% within the respective lifestyle. The relatively small number of taxa specific to non-industrialized samples may result from differences in sample size between the two lifestyles. Therefore, we created 1000 downsampled versions of the dataset in which the number of studies, individuals, and samples above and below 1 year of age were equal for both lifestyle groups. In the downsampled datasets, we still detected significantly more lifestyle-specific genera in industrialized microbiomes compared to non-industrialized ones, although the difference was less pronounced (mean = 267.0 and 52.4, median = 270 and 51, respectively, paired Wilcoxon test, *p* value < 0.001) (Supplementary Fig. S1B).

**Table 1:** General characteristics of the different studies included in the analysis

Study ID	Lifestyle	Country	No. samples	No. individuals
Beller_2021^72^	Industrialized	BELGIUM	233	8
Blanton_2016^73^	Non-industrialized	MALAWI	157	59
Bockulich_2016^8^	Industrialized	USA	806	43
Davis_2017^74^	Non-industrialized	GAMBIA	47	28
Gehrig_2019^24^	Non-industrialized	BANGLADESH	54	3
Hill_2017^7^	Industrialized	IRELAND	531	144
Kortekangas_2020^75^	Non-industrialized	MALAWI	1405	606
Kristensen_2020^76^	Industrialized	NETHERLANDS	502	45
Lim_2015^77^	Industrialized	USA	48	8
Morandini_2023^9^	Non-industrialized	SENEGAL	69	52
Muinck_2018^78^	Industrialized	NORWAY	2684	12
Pannaraj_2017^79^	Industrialized	USA	207	66
Raman_2019^23^	Non-industrialized	BANGLADESH, INDIA, PERU, SOUTH_AFRICA	1774	84
Raman_2019	Industrialized	BRAZIL	124	7
Reyman_2019^38^	Industrialized	NETHERLANDS	1030	120
Roswall_2021^26^	Industrialized	SWEDEN	1053	465
Sprockett_2020^80^	Non-industrialized	BOLIVIA	157	48
Stokholm_2018^81^	Industrialized	DENMARK	1280	617
Subramanian_2014^10^	Non-industrialized	BANGLADESH	210	25
Vatanen_2018^82^	Industrialized	FINLAND, ESTONIA, RUSSIA	2673	289
Wampach_2018^83^	Industrialized	LUXEMBOURG	84	18

We observed that industrialized samples had, on average, a two times higher sequencing depth compared to non-industrialized samples (Supplementary Fig. S1C). Therefore, we rarefied to 2000 reads per sample for alpha diversity analyses. We observed a positive relationship between the genus richness and the number of studies, individuals, and samples within each lifestyle, independently of the sequencing depth (Supplementary Fig. S1D–F). Overall, in any of the lifestyles, the number of detected genera reached the saturation of the community, suggesting an unexplored microbial diversity in infants from both industrialized and non-industrialized lifestyles. Genus richness was significantly lower in non-industrialized than in industrialized samples, regardless of the number of studies, individuals, and samples included in both groups (likelihood ratio-test (LRT), *χ*² (DF) = 1.72, 2.32, 4.45, *p* value < 0.01, explained variance: 8.7, 16.0, 15.6% for the number of studies, individuals, and samples, respectively).

**Fig. 1:**
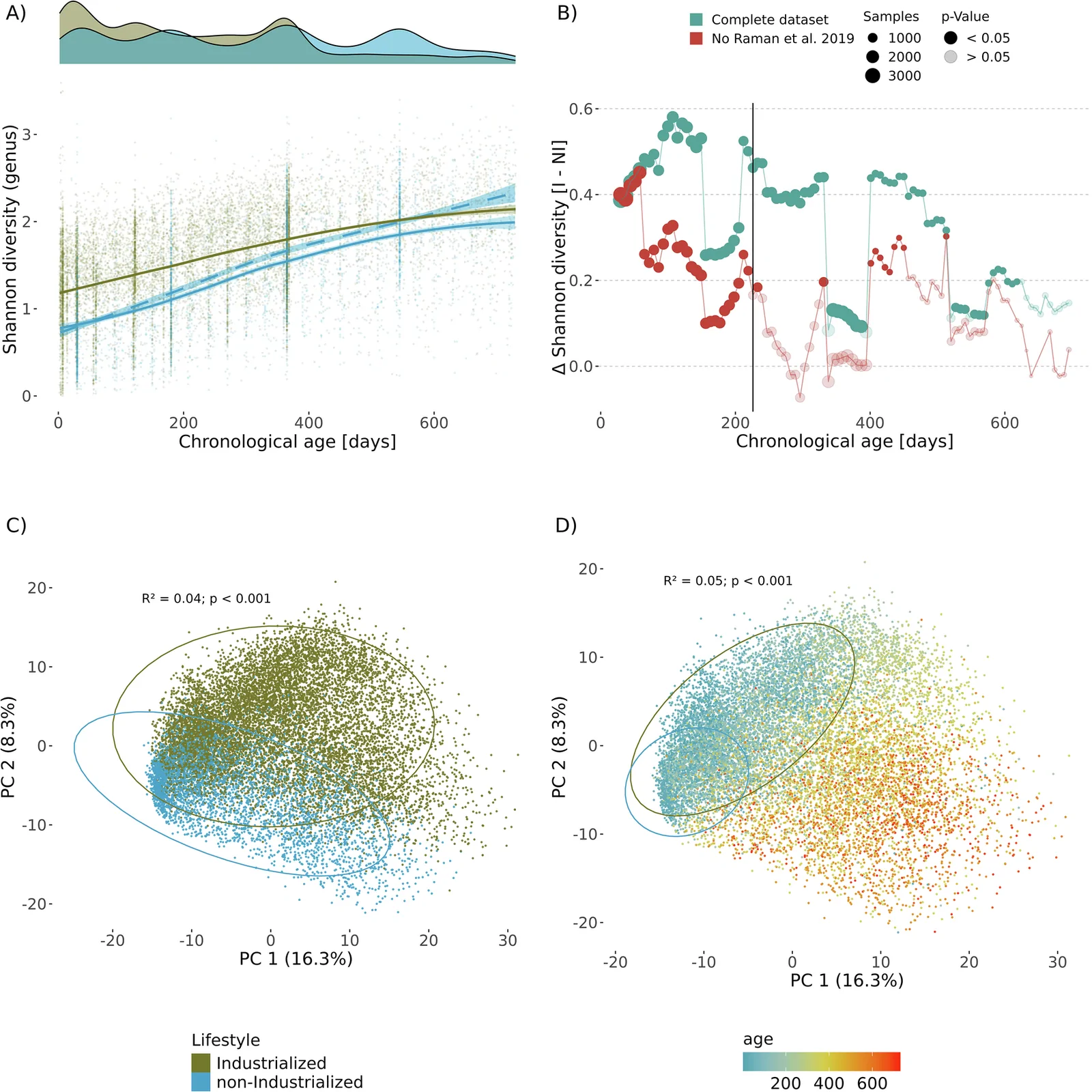
Gut microbial diversity and community structure follow lifestyle-specific maturation trajectories. **A** Shannon diversity calculated from rarefied counts aggregated to genus level, plotted over chronological age and stratified by lifestyle. Locally estimated scatterplot smoothing (LOESS) curves are fit to visualize temporal trends. The solid lines represent the independent increase in alpha diversity by lifestyle. The dashed line shows the increase in diversity for non-industrialized microbiomes, excluding one study with a large sample size (Raman et al.^23^). When all non-industrialized studies are included, diversity is reduced overall compared to industrialized studies throughout the entire chronological age span. However, when all samples from Raman et al.^23^ are removed, the difference in diversity is significant only during the first 200 days of life. **B** Difference in Shannon diversity between industrialized (I) and non-industrialized (NI) populations in 60 days sliding window. Red represents comparisons on the complete industrialized vs non-industrialized datasets, and blue comparisons between complete industrialized and a reduced non-industrialized dataset without the study of Raman et al.^23^. The number of samples in each comparison is represented by size. Transparent points indicate no significant differences between lifestyles (Wilcoxon test with Bonferroni correction for multiple testing). **C** Principal Component Analysis (PCA) on rarefied centered-log-ratio transformed counts colored by lifestyle and **D** age in days of life. *R*^2^ values indicate the proportion of variance explained in a Permutational multivariate analysis of variance (PERMANOVA) using a sequential model with the terms age, lifestyle, and study.

Microbial diversity (Shannon index, Fig. 1A) and richness (Supplementary Fig. S1G) increased along the chronological age (LRT, *χ*² (DF) = 0.85, *p* value < 0.01, explained variance: 18.8%), consistent with previous studies^5,8,22^. While the increase in alpha diversity metrics was independent of lifestyle, non-industrialized microbiomes showed reduced overall diversity compared to industrialized ones (LRT, *χ*² (DF) = 0.80, *p* value < 0.01, explained variance: 0.1%). The non-industrialized samples had a significantly lower fraction of amplicon sequence variants (ASVs) unassigned at the genus level compared to industrialized samples (LRT, *χ*² (DF) = 0.49, *p* value < 0.01, explained variance: 0.2%) (Supplementary Fig. S1H), indicating that bias in the annotation database is not the cause of a reduced alpha diversity in non-industrialized samples. A study by Raman et al.^23^ with 1891 mainly non-industrialized samples drove this significant effect on alpha diversity between lifestyles, especially at older ages. The variance explained by lifestyle was lower when this study was not included (LRT, *χ*² (DF) = 0.88, *p* value = 0.78, explained variance <0.001%). However, alpha diversity was consistently decreased for non-industrialized individuals within a 60-day sliding window from birth to until around 200 days of chronological age, regardless of whether the samples from Raman et al. were included (Wilcoxon test, Bonferroni-adjusted *p* value < 0.05, mean ΔShannon diversity: 0.43 before and 0.28 after 200 days of life including Raman et al.; 0.26 before and 0.11 after 200 days of life excluding Raman et al.) (Fig. 1B). The reduced diversity effect was primarily due to non-industrialized individuals from Bangladesh and South Africa from the Raman et al. study. However, individuals from Bangladesh in Gehrig et al. ^24^ show a similar trend, while those in Subramanian et al. ^10^ do not (Supplementary Fig. S1I). These results demonstrate the heterogeneity of the non-industrialized dataset and highlight the importance of gathering multiple studies to assess differences in alpha diversity between lifestyles that are not observable in single cohorts with restricted age spans.

A total of 4% of the microbial community structure was determined by the lifestyle (PERMANOVA on Euclidean distances from centered-log-ratio (CLR) transformation, *p* < 0.001), with a clear separation between non-industrialized and industrialized samples along PC2 (Fig. 1C, Supplementary Fig. S2A). Among the most influential taxa driving this separation are *Veillonella*, *Erysipelatoclostridium*, and *Bacteroides* in the industrialized direction and *Faecalibacterium* in the non-industrialized direction (Supplementary Fig. S2A). An additional 5% was explained by the age (PERMANOVA, *p* < 0.001), with the distinction between lifestyles becoming clearer at older chronological ages, following parallel trajectories along PC1 over time (Fig. 1D, Supplementary Fig. S2A). This trajectory was correlated with Shannon diversity and was mainly driven by *Faecalibacterium*, *Bacteroides*, *Lachnoclostridium, Intestinibacter*, and *Ruminococcus* (Supplementary Fig. S2A). Although the study-specific effects accounted for 7% of the residual variation (Supplementary Fig. S2B), there was a consistent influence of age and lifestyle. While the average sequencing depth differed between the two lifestyle groups, a PCA on rarefied counts showed the same pattern in sample distribution. The first two principal components were largely driven by age and lifestyle, respectively (PERMANOVA, *p* < 0.001, explained variance: 9% (age), 6% (lifestyle)), indicating a biological rather than a technical effect driven by sequencing depth difference (Supplementary Fig. S2C, D).

### A model for microbiome maturation in human infants

**Fig. 2:**
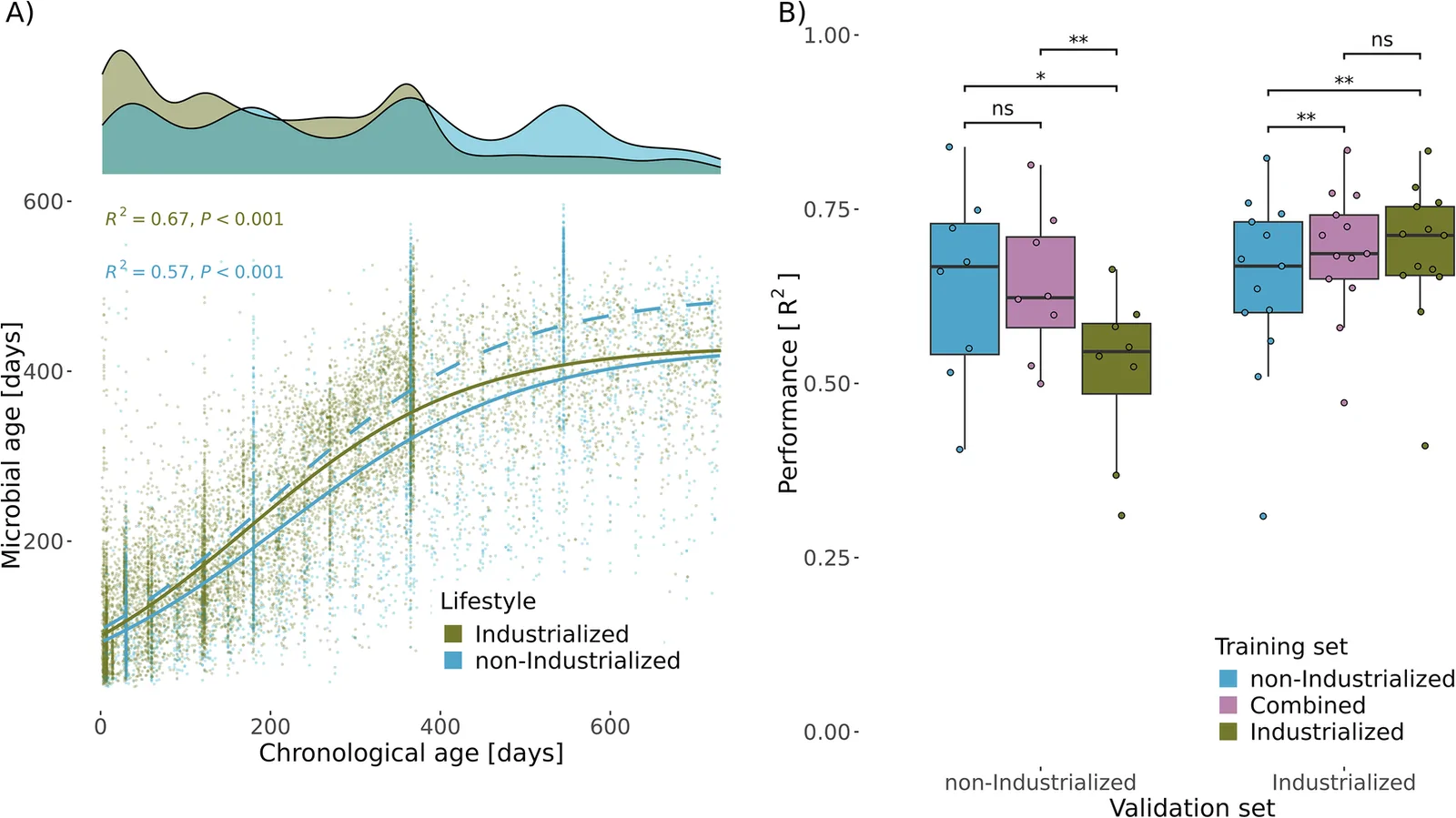
Lifestyle determines microbial age modeling but not development stages. **A** Microbial age development over chronological age. Microbial age was predicted using random forest regression based on relative abundances of bacterial genera against chronological age using a leave-one-study-out cross-validation (LODO-CV) approach. A logistic growth curve was fit to visualize the non-linear trajectory of the maturation dynamic over chronological age. The solid lines represent the microbial age by lifestyle with the complete datasets. The dashed line shows the microbial age in non-industrialized individuals, excluding the study by Raman et al.^23^. **B** Performance of microbial age modeling with lifestyle-specific models. Separate models were trained either on microbiomes from industrialized samples, non-industrialized samples, or a combined dataset. Performance was assessed using LODO-CV, where each study served as a validation set once for each model. Coefficient of determination (*R*^2^) from a linear model of rank-transformed microbial age versus chronological age was used as a performance metric and was calculated for each combination of model and validation set separately. Differences in model performances were tested with a paired Wilcoxon test and adjusted for multiple comparisons using Bonferroni correction (*padj < 0.1, **padj < 0.05, ***padj < 0.01).

We developed a microbiome age index by training machine learning models that predict an individual’s age based on their microbial composition and diversity at the time of sampling to identify microbial features that characterize the maturation process in different lifestyles. The model’s evaluation using leave-one-dataset-out cross-validation (LODO-CV) demonstrated a strong correlation between predicted microbial age and chronological age in both industrialized and non-industrialized samples (*R*^2^: 0.67 and 0.57, respectively), with the minimum estimated microbial age at 28 days (Fig. 2A, Supplementary Fig. S3A).

Microbial age initially increased with chronological age, reaching a stable phase at 455 and 524 days for industrialized and non-industrialized lifestyles, respectively (90% of carrying capacity, logistic growth model), following the pattern observed for microbial diversity. This reflects a phase of rapid initial microbial colonization directly after birth, followed by a more stable phase where the community reaches stability. Although the overall maturation trajectory remained consistent across lifestyles, non-industrialized infants exhibited a distinct maturation trajectory compared to industrialized infants (LRT, *χ*² (DF) = 1, *p* value < 0.01, explained variance = 0.1%). Similarly, as for alpha diversity, the 1891 samples from Raman et al. were highly influential on the distinct maturation trajectory, further confirming the high variability among non-industrialized maturation trajectories and difficulties of generalization among studies with non-industrialized lifestyles. While delivery mode is known to influence early infant gut microbiome development^6,7^, it only slightly impacted microbial age prediction (Supplementary Fig. S3B, LRT, *χ*² (DF) = 1, *p* value < 0.01, explained variance = 0.01%). We additionally evaluated the model’s performance across different age groups and cohorts using root mean square deviation (RMSE) values (Supplementary Fig. S3C). RMSE values were influenced by the non-linear relationship between microbial and chronological age. High RMSE values were observed at early ages (0–5 months) and late ages (>20 months) as a result of Random Forest bias at upper and lower values within the age range. For that reason, further analyses used *R*^2^ as a performance metric.

To determine whether microbial age prediction differs between industrialized and non-industrialized infants, we trained specific models on three types of datasets: (1) a combined dataset including samples from both lifestyles, (2) a dataset with samples from the industrialized lifestyle, and (3) a dataset from a non-industrialized lifestyle and evaluated their performance on lifestyle-specific datasets as validation (Fig. 2B, Supplementary Table S2, Supplementary Fig. S4A). Models trained on a combined dataset did not significantly outperform lifestyle-specific models when the lifestyle of the validation and training set matched. However, models trained on a single lifestyle and evaluated on a different lifestyle validation dataset showed a significant decrease in performance. The performance in microbial age prediction of non-industrialized samples using an industrialized model significantly declined (paired Wilcoxon test, Bonferroni-adjusted *p* value < 0.1) compared to models that included non-industrialized samples in the training set. For industrialized datasets, the performance in predicting the microbial age showed a significant reduction when using models trained only on non-industrialized samples (paired Wilcoxon test, Bonferroni-adjusted *p* value < 0.05). The findings remained consistent when the models were trained with the taxa agglomerated at the family level (Supplementary Fig. S4B).

To determine whether differences in sequencing depth had an effect on the observed differences between the two lifestyles, we rarefied the samples to the same sequencing depth and trained a new model on the rarefied dataset (Supplementary Fig. S4C). The significant differences in model performance between different lifestyles were reproducible. While the combined model did not perform significantly better than the industrialized model for non-industrialized data, the direction of the effect remained consistent. Studies including non-industrialized individuals were underrepresented in our study. To assess the effect of lower sample size on the performance of microbial age prediction of models trained on non-industrialized samples, we repeated the analysis for each validation set on 50 downsampled versions of the training set where both lifestyles had a similar number of studies, individuals, and samples above and below 1 year of age. We found a reproducible difference in model performance between lifestyles when the downsampled industrialized training sets were used (Supplementary Fig. S4D).

Non-industrialized samples represent a heterogeneous lifestyle group, defined by the absence of industrialized lifestyle characteristics rather than sharing a similar lifestyle. Living conditions include inhabitants of a slum in Bangladesh, as well as infants living in the Amazon rainforest. Despite the expected variability among non-industrialized samples, our results indicate that training models on a more diverse dataset improved their generalizability for all non-industrialized datasets.

### Specific bacteria drive the microbiome maturation in different lifestyles

**Fig. 3:**
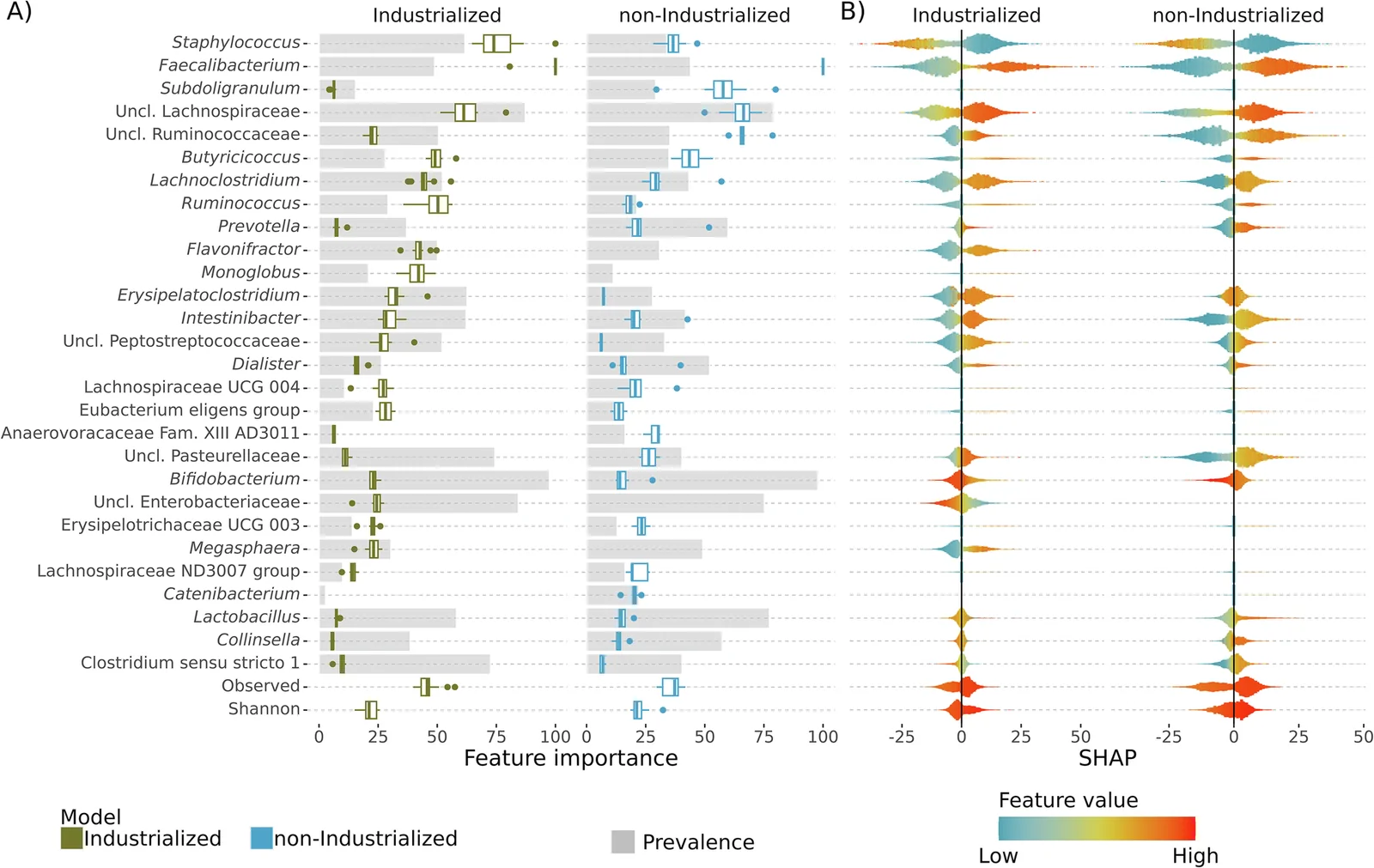
Specific bacterial taxa distinguish the microbial maturation between lifestyles. **A** Relative feature importance of specific bacteria is different depending on the lifestyle, and its relevance is independent of its prevalence. Relative feature importance in random forest models trained separately on samples from individuals with an industrialized or a non-industrialized lifestyle. Gray bars indicate the mean prevalence of a specific taxon across studies in both lifestyles. Importances are displayed only for features significantly important for the respective lifestyle. **B** SHapley Additive exPlanation (SHAP) values for specific bacteria show consistent predictive impact of a given feature between the two lifestyles. However, the abundance of *Lactobacillus* or *Collinsella* has the opposite effect on the prediction of microbial age between lifestyles: A higher abundance increases the predicted age in non-industrialized individuals, and decreases it in industrialized individuals. Violin plots represent the distribution of SHAP-values per feature. Positive SHAP-values indicate an increase in predicted microbial age driven by a given feature, whereas negative SHAP-values indicate a decrease in the predictive effect. Color indicates relative taxon abundance or alpha diversity, scaled per feature. Only values for features significantly important for the respective lifestyle are shown.

Having identified consistent differences in microbial maturation between lifestyles, we aimed to investigate which microbial taxa characterize these differences. Thus, we calculated the feature importance scores for the random forest models (Fig. 3A, Supplementary Fig. S5A, Supplementary Table S3). Overall, we identified 105 important taxa that account for a mean combined relative abundance of 98% across all samples. However, only 40 of those taxa were important in both lifestyles, and the majority were age informative in only one lifestyle. Among the generalist taxa shared across the lifestyles, *Faecalibacterium* was the most important taxon in both lifestyles. In contrast, *Ruminococcus* and *Staphylococcus* were more important in industrialized microbiota than in non-industrialized models, and *Subdoligranulum* and *Prevotella* showed greater importance in non-industrialized models, suggesting them as lifestyle specialists. Additionally, 65 taxa were important only for one lifestyle. *Monoglobus* and *Flavonifractor* were examples of taxa highly relevant only in industrialized microbiota, while *Oscillospiraceae UCG 002* was an exclusive specialist in non-industrialized individuals. All taxa important in only one lifestyle-specific model were still present in the other lifestyle, although less prevalent (Supplementary Fig. S5A). Feature importance was independent of prevalence for the non-industrialized dataset. For the industrialized dataset, however, feature importance was only correlated with prevalence for taxa with a prevalence below 0.15 (Supplementary Fig. S5B).

More taxa were classified as important in industrialized than in non-industrialized models (95 and 50, respectively). Our selection of important taxa could have been affected by the number of studies and, thus, the number of models in the LODO-CV for each lifestyle. Therefore, we compared the number of taxa identified as significantly important by Boruta in each lifestyle-specific model trained on the downsampled datasets described above. Industrialized models still had a significantly higher number of important taxa on average than non-industrialized models (mean = 104 and 93, median = 105 and 94, respectively; Wilcoxon test: *p* < 0.001) (Supplementary Fig. S4E).

### Taxonomic drivers of maturation differ in their colonization patterns across lifestyles

**Fig. 4:**
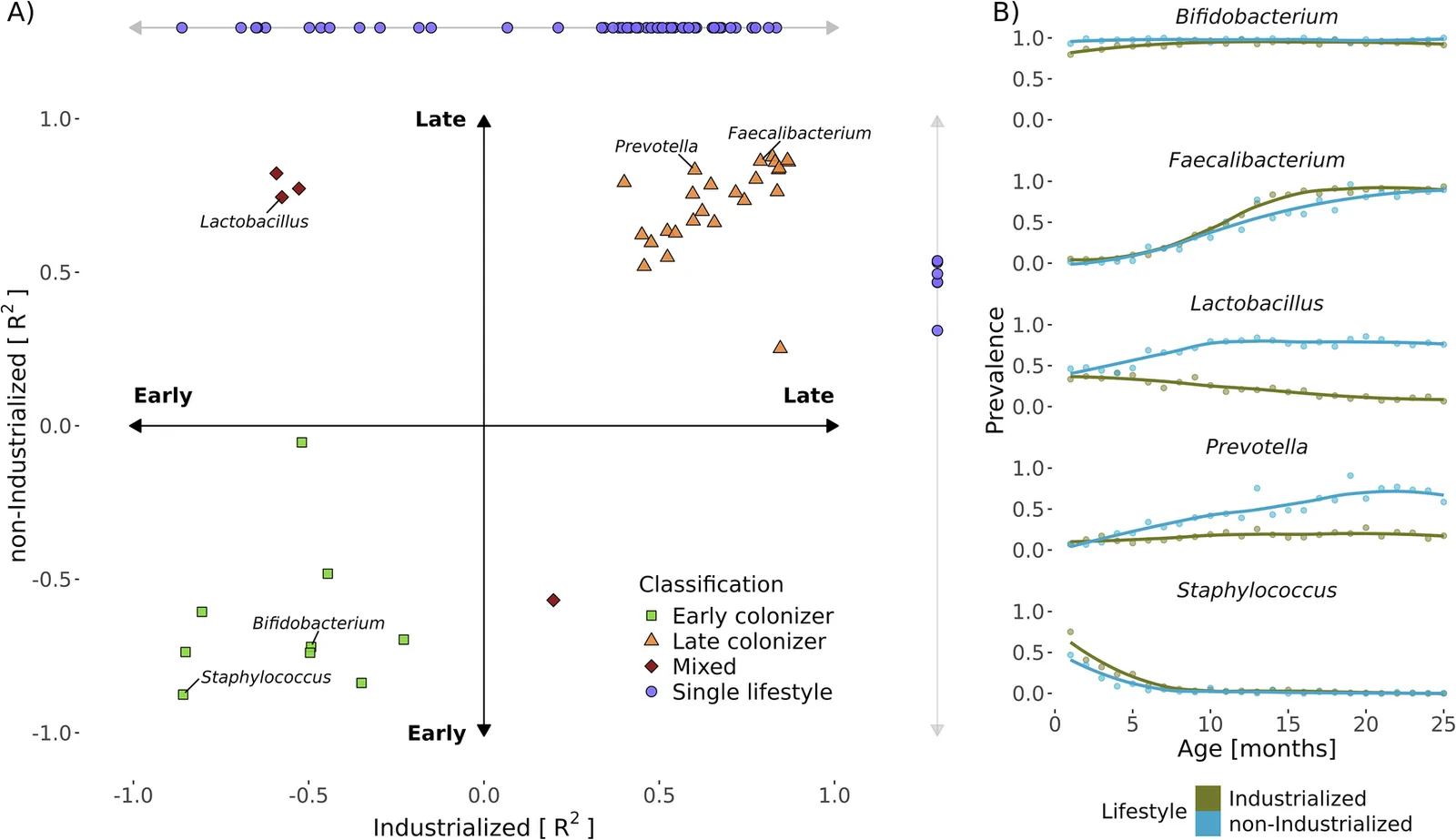
Bacterial taxa with significant global importance in lifestyle-specific models differ in their colonization dynamics. **A** The majority of taxa with high model importance in both lifestyles correspond to late (e.g., *Faecalibacterium* and *Prevotella*) or early (e.g., *Staphylococcus*) colonizers for both lifestyles. A set of taxa shows a mixed dynamic, meaning that colonization patterns differ between lifestyles. Spearman correlation between SHAP-values and relative taxon abundance in models trained on industrialized and non-industrialized samples for all significantly important taxa. Taxa consistently showing a negative correlation across both models are classified as early colonizers, while those with a positive correlation are classified as late colonizers. Taxa with discordant colonization patterns between lifestyles are classified as mixed, and those significantly important in only one lifestyle as single lifestyle. Correlations for taxa not significantly important in the respective lifestyle or with Bonferroni-adjusted *p* values > 0.05 are displayed on the top and right side of the plot. **B** Prevalence of taxa representative of the different colonization dynamics in samples from both lifestyles plotted over chronological age, binned in months.

Once we identified specialist and generalist features for both lifestyles, we aimed to further characterize the temporal dynamics of these features and define the changes in maturation between the two lifestyles. Therefore, we performed SHapley Additive exPlanation (SHAP) analysis to assess the contribution of each feature to the predicted microbial age^25^. Positive SHAP-values indicate that a feature in a given sample increases the predicted microbial age, whereas negative SHAP-values indicate a decreasing effect. The three taxa with higher relative feature importance values, *Staphylococcus*, *Faecalibacterium*, and one unclassified Lachnospiraceae taxon, also showed the highest range of SHAP-values, underlining their importance in our model (Fig. 3B, Supplementary Fig. S5B). In general, feature values and their corresponding SHAP-values exhibited a positive monotonic relationship across taxa, meaning that the increase in feature value corresponds to an increase in SHAP-values, and vice versa. However, taxa like *Bifidobacterium* and *Staphylococcus* showed negative relationships. While the relationship between feature values and SHAP-values was consistent across lifestyles for most taxa, *Lactobacillus* and *Collinsella* showed opposite relationships in each lifestyle group, indicating lifestyle-dependent ecological roles of those taxa. In the case of *Lactobacillus*, the effect is represented by the contrasting prevalence along the age (Fig. 4B). All predictive taxa had a significant difference in SHAP-value distribution between lifestyles (*p* < 0.001, Kolmogorov–Smirnov test, Bonferroni correction, Kolmogorov–Smirnov *D* = 0.26 between models, 0.35 within the industrialized model and 0.39 within the non-industrialized model) (Supplementary Fig. S6C), which further confirmed the difference in predictive importance for each taxa between lifestyles and within lifestyles.

We further evaluated the relationship between the predictive influence of each feature and the predicted microbial age and calculated Spearman correlations between feature abundance values and their corresponding SHAP-values. A positive correlation indicates that higher abundance values for a feature are associated with an increased predicted microbial age and vice versa. Based on the association between abundance and predictive influence of each taxon, we grouped taxa into four colonization patterns. Early colonizer taxa were more abundant in early-life and declined over time, whereas late colonizers were largely absent in the first months of life and increased in abundance later in life. Mixed colonizers include taxa whose colonization patterns differed between lifestyles, and single lifestyle taxa were only important in models from one lifestyle.

The majority (89%) of taxa important in both lifestyles had a consistent colonization pattern across lifestyles (Fig. 4A, Supplementary Table S3). Late colonizers accounted for 66% of these taxa. Similarly, the majority (79%) of the single lifestyle taxa exhibited a late colonizer pattern. These features combined drive the increase in alpha diversity described above (Fig. 1A) and the maturation of microbial communities with age.

*Faecalibacterium*, as an example of a late colonizer in both lifestyles, was nearly absent in the first months of life and increased in prevalence and abundance over time with a similar rate of increase in both lifestyles (Fig. 4B, Supplementary Fig. S7). *Prevotella* also displayed a late colonizer pattern in both lifestyles. However, in non-industrialized individuals, the rate of increase in prevalence was higher than for industrialized individuals (LRT, *χ*² (DF) = 1.00, *p* value < 0.001), indicating a different colonization dynamic between lifestyles. *Staphylococcus*, as an early colonizer, was predominantly detected during the first 7 months of life and declined in prevalence thereafter, while *Bifidobacterium* remained present in most individuals but decreased in relative abundance over time. *Lactobacillus*, classified as a mixed colonizer, initially had a similar prevalence in both lifestyles; however, while it declined in industrialized infants, it became increasingly prevalent in non-industrialized infants (LRT, *χ*² (DF) = 1.00, *p* value < 0.001).

### Health conditions affect microbiome maturation

We applied the combined and lifestyle-specific models to two independent datasets from clinically relevant conditions. One comprising infants diagnosed with severe acute malnutrition (SAM) from two cohorts in Bangladesh, considered as a non-industrialized cohort. The other consisted of preterm infants from three industrialized cohorts. All comparisons were made between both clinical conditions and healthy cohorts with both lifestyles. Both clinical conditions clearly represent distinct microbial communities from healthy, industrialized, and non-industrialized infants (Supplementary Fig. S8A, B). SAM infants consistently showed a reduced Shannon diversity compared to healthy non-industrialized infants (LRT, *χ*² (DF) = 0.25, *p* value < 0.01, explained variance: 2.3%), whereas preterms did not differ significantly from healthy full-term infants (LRT, *p* value > 0.1) (Supplementary Fig. S8C, D).

**Fig. 5:**
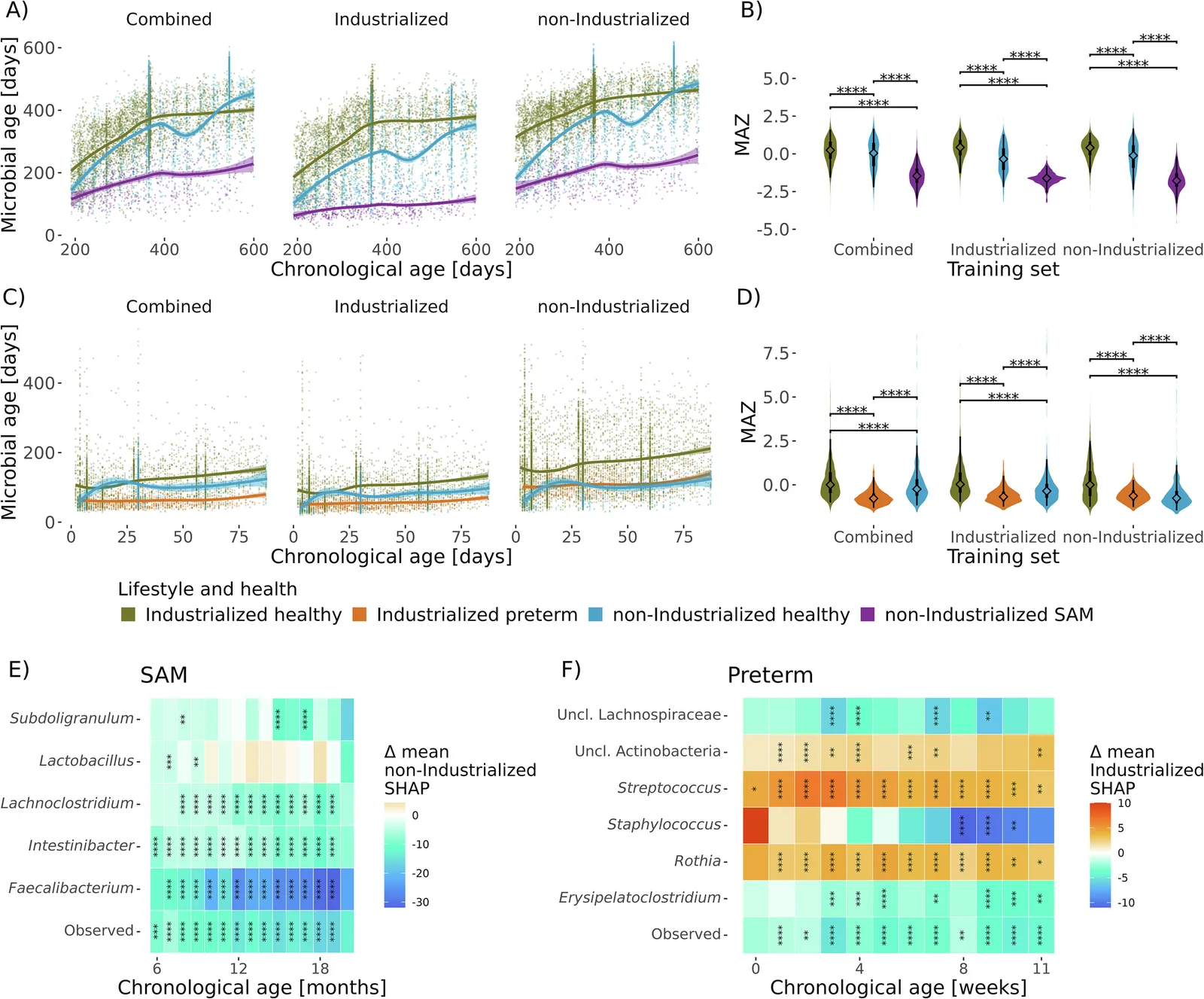
Health conditions affect microbiome maturation differently in lifestyle-specific models. **A** Microbial age development over chronological age in combined and lifestyle-specific models for healthy industrialized and non-industrialized infants and non-industrialized infants with acute severe malnutrition (SAM). LOESS curves are fit to indicate temporal trends. **B** Differences in microbiome-for-age *Z*-scores (MAZ) between SAM and healthy infants from both lifestyles predicted with a combined and the lifestyle-specific models. Diamonds represent medians, thick lines 50th percentiles, and thin lines 95th percentiles. Differences in MAZ were tested with a Wilcoxon test and adjusted for multiple comparisons using Bonferroni correction (*padj < 0.1, **padj < 0.05, ***padj < 0.01, ****p < 0.001). **C** Microbial age develo*p*ment over chronological age in combined and lifestyle-specific models for full-term healthy industrialized and non-industrialized infants and preterm-born industrialized infants. **D** Differences in MAZ between preterm and healthy full-term infants from both lifestyles predicted with a combined and the lifestyle-specific models. **E** Differences in mean SHAP-values between non-industrialized infants with SAM and healthy non-industrialized infants for selected taxa binned by month. SHAP-values are based on the non-industrialized model. Differences in SHAP-values were tested for each age bin with a Wilcoxon test and adjusted for multiple comparisons using Bonferroni correction (*padj < 0.1, **padj < 0.05, ***padj < 0.01, ****padj < 0.001). **F** Differences in mean SHAP-values between preterm and full-term industrialized infants for selected taxa binned by week. SHAP-values are based on the industrialized model.

SAM infants exhibited delayed microbial maturation compared to healthy infants from both lifestyle groups across models (Fig. 5A, B, Supplementary Fig. S9A–C), consistent with previous findings^10,24^. The difference in microbial age prediction for SAM infants and healthy non-industrialized infants was stronger when predictions were based on a model including non-industrialized samples (Δmean MAZ_non-industrialized_ = 1.44 and Δmean MAZ_combined_ = 1.40) compared to the industrialized (Δmean MAZ_industrialized_ = 1.15) model. To confirm that differences in age distribution did not bias the prediction, we predicted the microbial age based on the models trained with downsampling data where both lifestyles had a similar number of studies, individuals, and samples above and below 1 year of age (Supplementary Fig. S10A, B). The industrialized model underestimated microbial age for SAM and healthy non-industrialized infants, supporting the need for lifestyle-specific models to assess microbial age not only for healthy infants but also for those that deviate from healthy conditions.

To further investigate the main drivers of this delay, we calculated SHAP-values for healthy and SAM infants within the non-industrialized dataset based on the non-industrialized model. We identified 69 microbial features significantly associated with delayed maturation. The most influential were the abundance of *Faecalibacterium* and microbial richness (Fig. 5E, Supplementary Fig. S8E). When compared to healthy non-industrialized infants, SAM infants had very low levels of the important late colonizer *Faecalibacterium* and did not show any increase in microbial richness over time (Supplementary Fig. S9D).

Preterm infants showed delayed microbiome maturation compared to industrialized full-term infants, which persisted at least up to 3 months of age (Fig. 5C). The delay in maturation was larger in models including industrialized full-term infants (Δmean MAZ_combined_ = 0.93 and Δmean MAZ_Industrialized_ = 0.88) than in the non-industrialized model (Δmean MAZ_non-industrialized_ = 0.76). The non-industrialized model predicts increased microbial age in all preterms and, in general, industrialized infants. This resulted in lower MAZ in non-industrialized full-terms compared to industrialized preterms (Fig. 5C, D, Supplementary Fig. 9E). Similarly, as for SAM infants, we confirmed the lack of bias in microbial age prediction due to differences in chronological age distribution (Supplementary Fig. S10C, D). Although the non-industrialized model predicted similar maturation trajectories for industrialized preterms and non-industrialized full-terms, both groups showed a distinct compositional difference, with preterm infants clustering separately from healthy infants, independently from lifestyle (Supplementary Fig. S8B). In contrast, both the combined and industrialized models distinguished the maturation trajectories of preterm-born industrialized infants and non-industrialized infants.

To investigate the drivers of delayed microbial maturation in preterm infants, we calculated SHAP-values for preterm and full-term industrialized samples based on the industrialized model. We identified 14 microbial features associated with delayed maturation in preterm infants. Microbial richness was the most influential feature, which was reduced in preterms at all ages (Fig. 5F, Supplementary Figs. S8F and S9F). Unlike in SAM infants, we identified 17 microbial features associated with accelerated maturation in preterms. While for SAM infants the drivers had a consistent effect across chronological age, most of the drivers in preterm infants were more influential at specific time points during maturation. We detected a persistent increase in the abundance of *Staphylococcus* in preterm infants that led to a decreased microbial age only at older chronological ages. One caveat of analyzing preterm infants is the reduced microbial diversity and rapid temporal shifts at early time points in life.

## Discussion

In this study, we analyzed the maturation dynamics of the infant gut microbiome between industrialized and non-industrialized populations using machine learning models and a globally diverse dataset of more than 14,000 early-life microbiomes from 20 different countries. This comprehensive approach allowed us to detect significant lifestyle informative maturation signatures. Here, we highlight the importance of diverse datasets in microbiome research, demonstrating that more homogenous datasets predominantly based on industrialized populations significantly decrease model performance for non-industrialized populations. Our analysis included a particularly diverse non-industrialized dataset, consisting of infants living in vastly different conditions, including a slum in Bangladesh and the Amazon rainforest. Despite the wide diversity of living conditions within these groups, a greater lifestyle diversity significantly improved model performance for all non-industrialized lifestyles. This enabled us to identify both distinct microbiome maturation dynamics compared to those of industrialized populations, and characteristics generalizable between all lifestyles, as well as deviations from a healthy microbiome maturation in clinically relevant conditions such as SAM or preterm birth.

At the intra-individual level, age was the strongest driver of microbial diversity. It rapidly increased during the first months of life, followed by a stabilization phase around 18 months consistent across lifestyles and in line with previous studies^5,8,26,27^. While previous studies observed a higher alpha diversity in non-industrialized infants after 2 years^5,28^, in our study, alpha diversity was reduced in non-industrialized individuals compared to industrialized ones early in life. The higher alpha diversity in the industrialized infants contrasts with the effects seen in the gut microbiota of industrialized adults, which usually have lower microbial diversity due to long-term poor fiber diets^29^. Our results may reflect the impact of a higher prevalence of formula feeding^30,31^, processed foods, and medical interventions in high-income countries on infants^32,33^, which is associated with an increased microbial diversity before the introduction of solid food. The reduced available metadata related to feeding and delivery mode represents a limitation for our interpretation. Nevertheless, our findings highlight the need for caution when generalizing diversity trends in early-life microbiomes from understudied non-industrialized populations.

In contrast to the minimal impact on intra-individual diversity, lifestyle had a strong and age-independent effect on the inter-individual microbiome variation and taxonomic composition. These compositional differences underscore the importance of lifestyle as a primordial ecological factor shaping the early-life microbiota rather than geography as previously reported^34^. Additionally, the high heterogeneity of the gut microbiomes in non-industrialized datasets, driven by different ecological, nutritional, medical, and sociocultural contexts^21^, explains why in our models, fewer taxa were identified as uniquely associated with non-industrialized populations than with industrialized ones. However, this still allows for the distinction of maturation dynamics from the more homogenous industrialized lifestyle. Our findings emphasize that while the microbial maturation process follows a conserved longitudinal trajectory, its community diversity and composition at an early age are distinctly modulated by lifestyle. This provides the foundation for exploring the specific colonization patterns that differentiate these microbial trajectories, as well as for predicting microbial age.

Microbial age prediction models revealed distinct colonization dynamics shaped by lifestyle. In non-industrialized infants, *Prevotella* appeared as a relevant late colonizer with high predictive importance, aligning with its known underrepresentation in the industrialized adult gut microbiome^35^. Our findings suggest that the diminished prevalence of *Prevotella* in industrialized adults may result from early-life colonization patterns. Conversely, *Staphylococcus* was identified as a highly relevant early colonizer in industrialized populations, possibly reflecting its higher prevalence in infants born via c-section, which is more common in countries with higher HDI^36–38^. Regardless of delivery mode, lifestyle differences may be related to hospital-acquired microorganisms, which increase in non-traditional birth settings. While not directly related to *Staphylococcus*, the microbiome of industrialized infants is substantially influenced by strains acquired from the environment, like nurseries^39^, however, this is not exclusive to industrialized populations and can also occur in non-industrialized infants. This indicates the influence of the local environment on infant microbiome colonization. Interestingly, while *Prevotella* and *Staphylococcus* showed consistent trajectories across lifestyles, *Lactobacillus* had divergent colonization patterns, increasing in prevalence in non-industrialized infants but declining over time in industrialized ones. Given the strong influence of exclusive breastfeeding on the infant gut microbiota^40^, differences in breastfeeding practices that have been documented in different populations^41^ might be additional factors related to divergent *Lactobacillus* patterns. Additionally, the observed contrasting patterns may reflect both ecological succession events during the maturation^42^ and differences in the prevalence of c-sections^3,36^.

Contrary to our hypothesis, not all the taxa known to play important roles in the early-life microbiota were informative for age prediction. Despite its well-documented biological role in the metabolism of breast milk oligosaccharides^43,44^, *Bifidobacterium* had little contribution to the performance of our microbial age prediction model, likely due to its ubiquity in infants. Higher taxonomic resolution has shown that lifestyle impacts the relative proportions of at least four different *Bifidobacterium* species^34,45^, which are not possible to distinguish with amplicon-based data. Furthermore, several genera that were identified as important for microbial age modeling in infants using shotgun data^12^, such as *Dorea*, *Blautia*, *Fusicatenibacter*, and *Agathobaculum*, were rarely detected in our dataset. This highlights a key challenge in using amplicon-based data for age modeling and emphasizes the need for higher taxonomic resolution to disentangle the functionality of distinct species or strains at different stages of an infant’s life^43,45^.

The overall performance of our modeling approach is slightly lower than that of previously published microbial age models^8,10,11,22,46^ (*R*^2^ = 0.72–0.82). However, those models were built and tested on a single cohort and thus lack generalizability to other populations. A modeling approach similar to ours, based on shotgun data from multiple cohorts and lifestyles^12^ achieved comparable performance (*R*^2^ = 0.64). While models on shotgun data do not outperform models on 16S data, the taxa important for age predictions differ. For example, *Blautia* and *Roseburia* were exclusively age-associated in shotgun-based models. *Faecalibacterium* is one of the most important taxa in most models, and *Bifidobacterium* is a common feature also detected by other models, although it differs in the rank of importance. *Ruminococcus* and *Staphylococcus* are highly important features in our model and others, but not in all of them. *Subdoligranulum* is highly important for our model, but not commonly detected in others.

Our modeling framework can detect clinically relevant deviations in microbiome maturation by applying it to infants diagnosed with SAM and preterm-born infants. Delayed microbiome maturation in SAM infants and microbial signatures distinguishing preterm from full-term infants have been reported previously^10,47^. However, our work demonstrates the applicability of this modeling approach to other infant-relevant clinical conditions, such as severe malnutrition and preterm birth, which complements the scope of previous microbiome age-lifestyle analysis^34^. Here, we identify informative microbial taxa associated with delayed maturation in both clinical conditions, providing a deeper insight into the drivers of these conditions. It has also been reported that malnutrition during pregnancy is associated with preterm birth^48^ and preterm-born infants show increased susceptibility to malnutrition^49^, particularly in low-income countries^50^. Our results indicate a potential developmental and nutritional link between the two conditions. Although the preterm and SAM infants differed in lifestyles and age span, further studies should examine whether delayed microbiome maturation represents a shared signature of both conditions. Notably, the observed differences in maturation delay between lifestyle-specific models highlight the importance of using reference populations that reflect the diversity of the target population.

Using our modeling framework based on amplicon data may limit interpretations due to biological and technical factors. One technical aspect is the rarefaction threshold that we implemented to maximize the number of samples included across the entire age spectrum in our analysis, while also controlling for uneven sequencing depth. However, using only the sequencing depth necessary to approach saturation as the basis for the rarefaction threshold may not accurately represent the diversity of fully mature microbiomes in older children, who may not have reached an asymptote yet. This approach results in the underrepresentation of extremely rare microbes^51^. A biological consequence is the taxonomic resolution. Bacteria such as *Dorea*, *Blautia*, *Fusicatenibacter*, and *Agathobaculum* have been reported as relevant for microbial maturation^12^, yet they were extremely low in prevalence (less than 0.1%) in our study, so our models did not detect them as relevant features for microbial age prediction. This could potentially be related to primer bias, which may not capture these specific genera. This relevance is generally better captured when shotgun metagenomic data is used^52^.

Despite the limited taxonomic resolution of amplicon-based data, our study advances our understanding of global microbiome maturation and highlights the value of integrating underrepresented populations into microbiome research. By compiling one of the most diverse infant microbiome datasets to date, we improved microbial age prediction models that can be applied across lifestyles and help address persistent geographic biases in the study of early-life microbiomes, and are comparable to other microbial age models. Previous work has shown that microbiomes from non-industrialized populations also differ markedly between those populations^53^. However, limited data from non-industrialized infant cohorts prevented deeper comparisons across lifestyles or larger geographic regions. One of the major advantages of our study is that it substantially increases the number of cohorts. This increase in the dataset was helpful to confirm the main findings from other studies^34^, but also to increase the number of individuals along the age range, diversity, and highlight the heterogeneity of both the non-industrialized and industrialized infants. Additionally, our work tests and evaluates the impact of lifestyle-specific models on microbial age prediction and its biological interpretation. Expanding studies of infant microbiome maturation in globally diverse populations will be essential to refine these models and broaden their applicability. Achieving more equitable and globally represented early-life microbial studies will also require going beyond sample inclusion to foster close collaborations and promote capacity building with local researchers and ensure reciprocal benefits for the communities^54^. While these goals go beyond the scope of this study, our work illustrates the potential and responsibility of microbial research to draw more globally representative conclusions.

## Methods

### Data collection

We collected publicly available 16S rRNA gene sequencing data from the stool microbiome of human infants. The inclusion of the samples in our meta-analysis was based on the following characteristics: (a) Samples from full-term, healthy infants under 2 years of chronological age, not subject to any intervention. (b) The sequencing data were generated using Illumina platforms. (c) The targeted amplicon included the V4 region of the 16S rRNA gene. (d) Metadata with information on the age of the infant at sampling time was available.

**Fig. 6:**
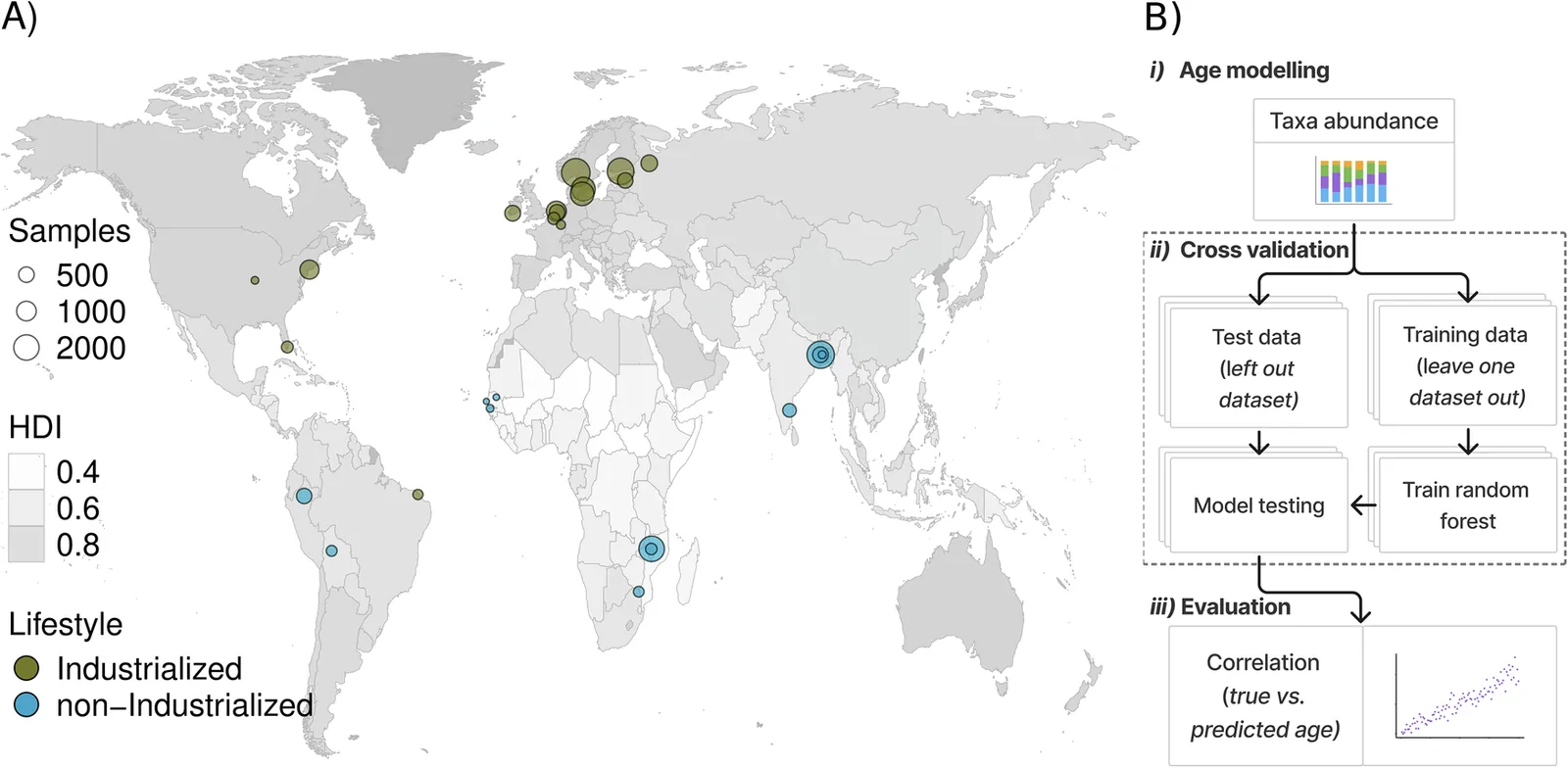
Collection and processing of publicly available datasets. **A** Global distribution of samples. Each circle represents the samples from one study at the specific location. Size indicates the number of samples, and color indicates the lifestyle of the samples. Shading indicates Human Development Index (HDI). **B** Computational pipeline for microbial age modeling. The processing has three steps: (i) raw data preprocessing and taxonomy annotation, (ii) random Forest regression models with leave-one-dataset-out cross-validation, and (iii) evaluation of predicted microbial age by correlation to chronological age.

Raw sequencing data were retrieved from the Sequence Read Archive, and metadata were obtained either from the original publications or through direct communication with the authors when necessary. The final dataset consisted of 14,979 samples from 20 studies across 20 countries in Africa, America, Asia, and Europe, corresponding to 2746 individuals (Fig. 6A, Table 1, Supplementary Table S1). Information on the delivery mode was considered from each study. However, this information was available only for 12 of the studies, which reduces the covariates included in the analysis. Age was considered in days since birth. For five studies, the age information was originally available in months and was converted to days by multiplying by a factor of 30. We categorized samples into two lifestyle groups: Industrialized and non-industrialized, using the Human Development Index 2022 (HDI)^55^ (Fig. 6A). Samples from countries with an HDI-value above the median of 0.742 were classified into the industrialized lifestyle, while all other samples were classified into non-industrialized lifestyles. Samples from Peru (HDI = 0.762) were treated as an exception and classified as non-industrialized, as they were taken from individuals living in a remote area of the Amazon rainforest with living conditions more comparable to other non-industrialized individuals than to industrialized ones^23^. The term industrialized lifestyle serves here as an umbrella term for complex, multidimensional lifestyle changes compared to pre-industrial societies, as previously discussed^21^. These changes include improved hygiene and sanitized environments, increased access to healthcare, and higher exposure to antibiotics and other drugs, reduced contact with wildlife, and a dietary shift toward more processed, high-caloric foods. These factors are known to have a huge impact on the human gut microbiome. While the use of HDI does not comprehensively represent the vast diversity comprising the “non-industrialized” category, it provides a non-arbitrary indicator for classification and highlights that our current view of infant microbiomes is biased.

Samples classified as industrialized in this study are expected to be more affected by these changes than those classified as non-industrialized. However, it is important to note that the term non-industrialized here does not refer to a single lifestyle, but rather to a collection of different lifestyles.

### Processing of sequencing data

The raw data were re-processed from quality check to taxonomic annotation with the same pipeline to ensure consistency across samples. The read quality was assessed with FastQC v0.11.9^56^. Where necessary, adapter contamination was removed from the sequences using Trimmomatic v0.39^57^. As a host decontamination step, reads were mapped to the reference human genome assembly (GRCh38.p14) using bowtie2 v2.3.4.3^58^ with the --very-sensitive option. Reads mapping to the human reference were removed from further analysis. Quality-checked and decontaminated reads were processed and merged to infer ASVs using the pipeline from DADA2 v1.34.0^59^ for each study independently. For study-specific details on mean, maximum, and minimum sequencing read depth for each study (Supplementary Table S1), trimming and filtering of reads. Taxonomic annotation of the ASVs was done with the SILVA database v138^60^ using DECIPHER v3.2.0^61^. ASVs assigned as “Mitochondria” were also discarded from further analysis. Different primer sets used in the studies (Supplementary Table S1) may lead to incompatible sets of ASVs, and for that reason, the dataset was aggregated to genus level for further analysis at the cost of losing resolution provided by the sequence variants. Based on rarefaction curves, study-specific thresholds were established to discard samples with low read counts (Supplementary Fig. S11, Supplementary Table S1). The filter threshold was determined as the number of reads where genus richness (number of observed genera) reached an asymptote. Relative abundances were calculated for the filtered datasets. Genera with a mean relative abundance below 0.005% were removed from all analyses. The bioinformatic analysis was performed on the High-Performance Computing Cluster from the Max Delbrück Centrum, Berlin (Max-Cluster).

### Alpha and beta diversity analyses

All analyses were run in R v4.4.2. Alpha diversity indices were estimated for all samples with more than 2000 reads after rarefication to that depth. At this depth, 99% of the samples had at least 2000 taxonomically assigned reads (Supplementary Fig. S11). For samples with less than 2000 reads that passed the study-specific filter threshold, alpha diversity indices were estimated based on the raw read counts. To evaluate whether differences in alpha diversity between lifestyles could be influenced by unevenness in sample sizes, the change in diversity with increasing dataset size was estimated. For each lifestyle group, random subsets were created by progressively increasing the number of studies, individuals, and samples. Subsamples were generated by increments of one study, 10 individuals, and 100 samples. For each number of studies, subjects, and samples, the mean number of taxa with more than 10 rarefied reads in at least one sample was determined out of 10 repetitions for each lifestyle group. Differences in the number of detected taxa between lifestyles over the number of studies, individuals, and samples were tested with generalized additive models, which capture non-linear relationships, using gamlss v5.4-22^62^. The number of taxa was modeled as a function of lifestyle, and a penalized spline smoothing term for the number of studies, individuals, or samples was included. The full model was compared with a reduced model without lifestyle using an LRT.

The effects of age and lifestyle on Shannon diversity were tested with generalized additive models. The full model had Shannon diversity as the response variable and lifestyle and age as explanatory variables, allowing for non-linear relationships between age and diversity using penalized spline smoothing. The study was included as a random factor. The full model was compared to two reduced models using an LRT, one without age and one without lifestyle, respectively. The proportion of variance explained by age and lifestyle was calculated as the relative decrease in deviance in the full model compared to the reduced model. The effect of individual studies on differences in Shannon diversity and the variance explained by each variable was assessed by repeating the analysis and removing each study from the dataset once. We compared the Shannon diversity of both groups using a 60-day sliding window advancing in 7-day increments to determine the age intervals in which microbial diversity differs significantly between the two lifestyles. We used Wilcoxon tests with Bonferroni correction for multiple testing.

For beta diversity analysis, a principal component analysis was computed on CLR transformed raw read counts and on CLR-transformed counts rarefied to 2000 reads per sample to assess the effect of differences in sequencing depth. The effects of age, lifestyle, and study on the composition were analyzed with a permutational analysis of variance (PERMANOVA) as implemented in vegan v2.6-8^63^. PERMANOVA was run with 999 permutations to model the Euclidean distance of the CLR-transformed count matrix by adding the terms age, lifestyle, and study sequentially. Other relevant factors, like delivery mode and breastfeeding, were not included in the analysis due to limited availability for all the studies.

### Machine learning modeling of age based on microbial composition

To predict the age of the individuals based on their microbial composition, a supervised machine learning model was trained. Random forest regression models with 500 trees were trained on a dataset including: (1) relative abundances of genera that were detected in at least five samples in two studies in the training set, and (2) microbial diversity (Shannon index) and richness. The models were trained on relative abundances to account for differences in sequencing depth between different studies and to allow future application of the models to other cohorts, independent of their sequencing depth. Random forest regression was implemented using the ranger R-package v0.17.0^64^ with default parameters. Relative feature importance was calculated for each feature as the increase in out-of-the-bag error when the respective feature was permuted and normalized to the highest importance value. Significantly important features were selected using the Boruta R-package v8.0.0^65^ with permutation-based importance values and a *p* value threshold of 0.01. To estimate the performance of the microbial age model, a LODO-CV was implemented using the caret R-package v6.0-94^66^. For this procedure, each study was left out one time as a validation set. The overall performance of the modeling approach was estimated using the predicted age from all cross-validation runs, eliminating the bias of different age distributions. Due to the non-linear but monotonous increase in microbial age alongside chronological age, microbial age was rank-transformed for model performance evaluation. A linear model between transformed predicted microbial and the actual chronological age was used to calculate the coefficient of determination (*R*^2^). The RMSE was also calculated as an additional performance metric (Supplementary Table S2, Supplementary Fig. S4). To assess the overall effect of lifestyle on the predicted microbial age obtained from the LODO-CV, a mixed model with the rank-transformed predicted age as response, chronological age, and lifestyle as explanatory variables, and study as a random factor was fit to the whole dataset. The full model was compared to a reduced model, without lifestyle, using an LRT. To assess the effect of individual studies on the differences in predicted microbial age, the same analysis was repeated by removing each study from the dataset once. To determine the time at which maturation reaches a more stable state, a logistic growth model was fitted to the relationship between chronological and predicted age for each lifestyle. We considered the time when the fitted model reached 90% of the model’s carrying capacity as the point at which the maturation rate decreased and reached a more stable state.

For lifestyle-specific models, only subjects from the corresponding lifestyle were used in the training set. The performance of lifestyle-specific models was compared across studies from both lifestyles using paired Wilcoxon tests with Bonferroni correction for multiple testing. Lifestyle-specific relevant features were defined if they were selected as relevant by Boruta in at least two models of LODO-CV for the specific lifestyle.

To assess the effect of differences in dataset size between the two lifestyles, an additional downsampling approach, independent from the one used for alpha diversity, was implemented. Lifestyle-specific and combined LODO-CV was performed 50 times for each study, with the training set downsampled to make the two lifestyles comparable in terms of the number of studies, individuals, and samples, as well as their age distribution. First, studies and individuals in the industrialized group were randomly selected in equal numbers to those in the non-industrialized group. Then, samples above and below 1 year of age were randomly downsampled separately to match the size of the smaller lifestyle group.

To determine the influence of important features on the prediction of age by lifestyle, two models were trained on the complete set of samples corresponding to each lifestyle. SHAP values were calculated for all samples on these two models using fastshap v0.1.1^67^. Kolmogorov–Smirnov test with Bonferroni correction for multiple comparisons was used to determine significance and effect size from differences in taxon-specific SHAP-value distribution between lifestyles and between taxa within specific lifestyles. The Spearman correlation between SHAP-values and relative feature abundances for each taxon was used to assess its temporal dynamics. Unlike the direct correlation between taxon abundance and age, the correlation between SHAP-values and abundance is a more robust strategy. It is less sensitive to zero-inflated abundance distributions and non-linear abundance-age relationships.

The longitudinal dynamics of taxa associated with lifestyle-specific maturation were further investigated to detect differences between the two lifestyles. Differences in trajectories of prevalence over time for those taxa were analyzed using linear models. Therefore, a full model was trained with age, lifestyle, and their interaction as predictors for prevalence. The full model was tested against a reduced model without the interaction between age and lifestyle as a predictor using an LRT as implemented in the lmtest R-package v0.9-40^68^.

### Influence of clinical factors on maturation

To evaluate the effect of lifestyle-specific models on the prediction of microbial age under two clinical conditions, we assembled two supplementary datasets which were not included in the training of any model: one from infants born preterm^69–71^ (before 37 weeks of gestation) and another from infants diagnosed with SAM^10,24^ (Supplementary Table S1). The raw sequences from these samples were processed as described above for the training set.

We predicted the microbial age of SAM and preterm infants employing the lifestyle-combined and lifestyle-specific models. To compare differences in maturation, microbiome-for-age *Z*-scores (MAZ) were calculated from microbial age predictions for each model. Microbial age predictions were grouped into monthly and weekly chronological age bins for SAM and preterm infants, respectively. Different bin sizes were chosen for SAM and preterm infants due to different age ranges of the available data. MAZ-scores were calculated by subtracting the group mean from the microbial age and dividing by the group standard deviation. To assess the influence of variable sample sizes in each bin on MAZ calculation, MAZ was additionally calculated based on the mean and standard deviation of 50 randomly chosen healthy samples per age bin. Differences in MAZ-scores between healthy, preterm, and malnourished infants were tested using Wilcoxon rank-sum tests with Bonferroni correction for multiple comparisons.

To determine microbial drivers of age maturation under different clinical conditions, SHAP-values were calculated for preterm and healthy industrialized infants using the industrialized model and SAM and healthy non-industrialized infants using the non-industrialized model. Relative taxon impact was assessed by the differences in the mean of SHAP-values between healthy infants and infants of the same lifestyle with a clinical condition in separate age bins using Wilcoxon tests. *P* values were adjusted for multiple testing across all taxa and age bins within each lifestyle group using Bonferroni correction.

For all the statistical analysis significance thresholds were defined at *p* = 0.05 and *p*-adjusted = 0.1.

## Supplementary information

Supplements-Global_analysis_of_infant_gut_microbiota-npjBM-(not-highlighted).

Supplementary_table_S1.

Supplementary_table_S2.

Supplementary_table_S3.

## Data Availability

All raw sequence data for the analysis are publicly available and were downloaded from the NCBI Sequence Read Archive. All the BioProjects included in this study are indicated in Table S1.

## Code availability

All the scripts and documentation from the analysis are available at (https://github.com/robbueck/microage).
