# Supplementary material for: Global analysis of infant gut microbiota revealed distinctive maturation dynamics across lifestyles
Source: NPJ Biofilms Microbiomes. 2026 Aug 19;12:162. doi: 10.1038/s41522-026-01130-4 (PMC13490558; doi:10.1038/s41522-026-01130-4)
Supplement: Supplementary file 1 [file 41522_2026_1130_MOESM1_ESM.pdf]

Global analysis of infant gut microbiota revealed distinctive maturation dynamics across lifestyles

Robert Bücking[1,2,3,4], Greta Pasquali[1,2,3,5], Ulrike Löber[1,2,3], Claudia Buss[6,7,8,9], Dorothee Viemann[4,10,11], Víctor Hugo Jarquín-Díaz[1,2,3]*, Sofia Kirke Forslund-Startceva[1,2,3,12,13]*

[1] Max-Delbrück Center for Molecular Medicine in the Helmholtz Association (MDC), Berlin, Germany
[2] Charité–Universitätsmedizin Berlin, a corporate member of Freie Universität Berlin and Humboldt-Universität zu Berlin, Berlin, Germany
[3] Experimental and Clinical Research Center (ECRC), a cooperation of the Max-Delbrück Center and Charité–Universitätsmedizin, Berlin, Germany
[4] Translational Pediatrics, Department of Pediatrics, University Hospital Würzburg, Würzburg, Germany
[5] University of Padua, Department of Medicine DIMED, Padua, Italy
[6] Charité–Universitätsmedizin Berlin, Corporate Member of Freie Universität Berlin, Humboldt-Universität zu Berlin, and Berlin Institute of Health (BIH), Institute of Medical Psychology, Berlin, Germany
[7] University of California, Irvine, Development, Health and Disease Research Program, Irvine, CA, USA
[8] German Center for Child and Adolescent Health (DZKJ), partner site Berlin, Charité - Universitätsmedizin Berlin, Germany
[9] German Center for Mental Health (DZPG), partner site Berlin, Charité - Universitätsmedizin Berlin, Germany
[10] Center for Infection Research, University Würzburg, Würzburg, Germany
[11] Cluster of Excellence RESIST (EXC 2155), Hannover Medical School, Hannover, Germany
[12] German Center for Cardiovascular Research (DZHK), partner site Berlin
[13] Structural and Computational Biology Unit (SCB), EMBL Heidelberg, Germany

*Corresponding author(s): Sofia Kirke Forslund-Startceva & Víctor Hugo Jarquín-Díaz. Max-Delbrück Center for Molecular Medicine (MDC), Experimental and Clinical Research Center (ECRC) Charité, Campus Buch. Lindenberger Weg 80, 13125, Berlin, Germany.
E-mail(s): Sofia.Forslund@mdc-berlin.de; VictorHugo.JarquinDiaz@mdc-berlin.de

Supplementary Data

Table S1. List of studies with number of samples and individuals, assigned lifestyle, countries of origin and filtering thresholds

Table S2. Classification of taxa into early and late colonizers for each lifestyle

Table S3. Model Performance

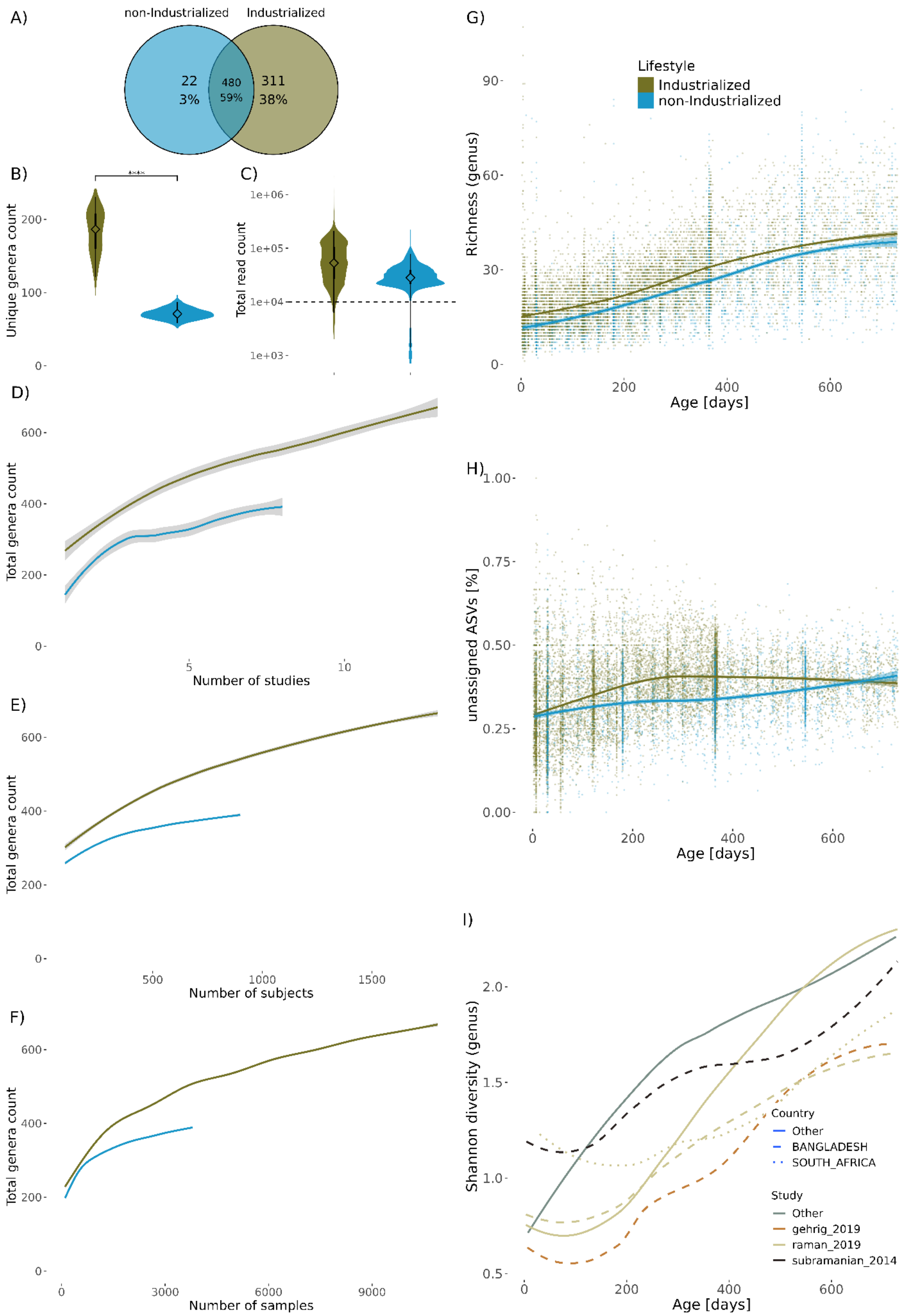

Supplementary Figure S1. Gut microbial diversity across lifestyles and datasets A) Venn diagram for the number of genera detected in each lifestyle. **B)** Difference in the number of genera detected only in industrialized or non-industrialized samples. Each value represents

one of 1,000 downsampling iterations. Diamonds represent medians, thick lines 50th percentiles, thin lines 95th percentiles. Significance was tested with a paired Wilcoxon test (****p<0.001). **C)** Difference in sequencing depth between lifestyles. Violin plot with the distributions of total read counts per sample. The dashed line marks 10,000 reads. Diamonds represent medians, thick lines 50th percentiles, thin lines 95th percentiles. **D, E, F)** Number of taxa detected in rarefied count data by lifestyle in randomly subsampled datasets to specific numbers of studies, subjects and samples. **G)** Microbial richness over time calculated from rarefied counts aggregated to genus level, plotted over chronological age and stratified by lifestyle. Locally estimated scatterplot smoothing (LOESS) curves are fit to visualize temporal trends. **H)** Fraction of ASVs unassigned at genus level per samples plotted over chronological age and stratified by lifestyle. Locally estimated scatterplot smoothing (LOESS) curves are fit to visualize temporal trends. **I)** Comparison of Shannon diversity between individuals from Bangladesh and South Africa and non-industrialized individuals from other countries. Shannon diversity was calculated from rarefied counts aggregated to genus level, plotted over chronological age and stratified by lifestyle. Locally estimated scatterplot smoothing (LOESS) curves are fit to visualize temporal trends.

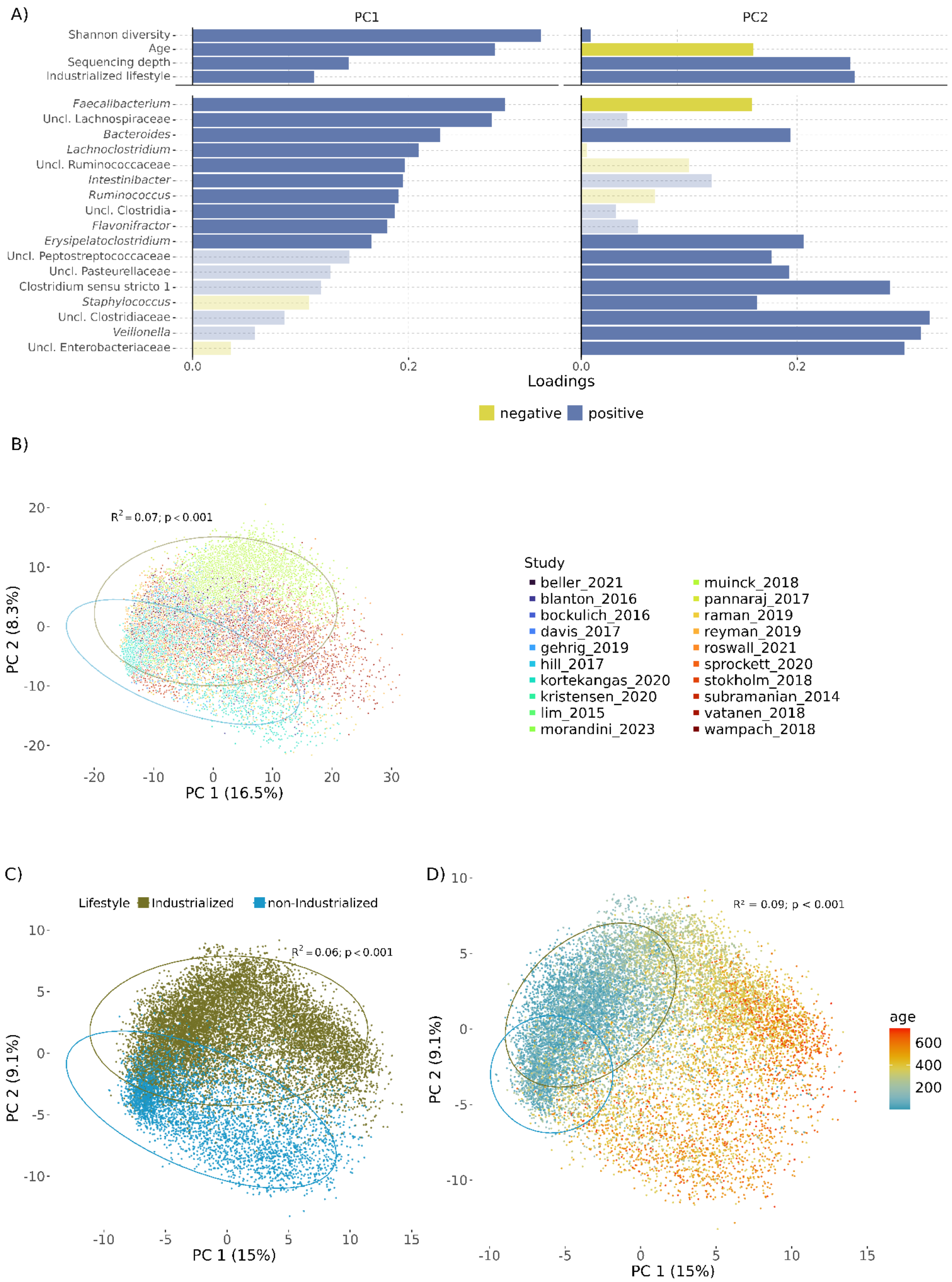

Supplementary Figure S2. Beta diversity across lifestyles and datasets A) Drivers of beta diversity in a PCA on CLR-transformed raw counts. Upper panel: Spearman correlations of sample variables with the first two principal components (PCs). Lover panel: Top 10 loadings of taxa on the first two PCs. Loadings not in the top 10 for one PC are transparent. **B)** PCA on CLR-transformed raw counts colored by study. R^2 value indicates the proportion of variance

explained in a PERMANOVA using a sequential model with the terms age, lifestyle and study. **C)** PCA on CLR-transformed rarefied counts colored by lifestyle and **D)** age. Read counts were rarefied to 2,000 per sample. Ellipses represent the 95% confidence intervals of lifestyle clusters in the reduced-dimensional space.

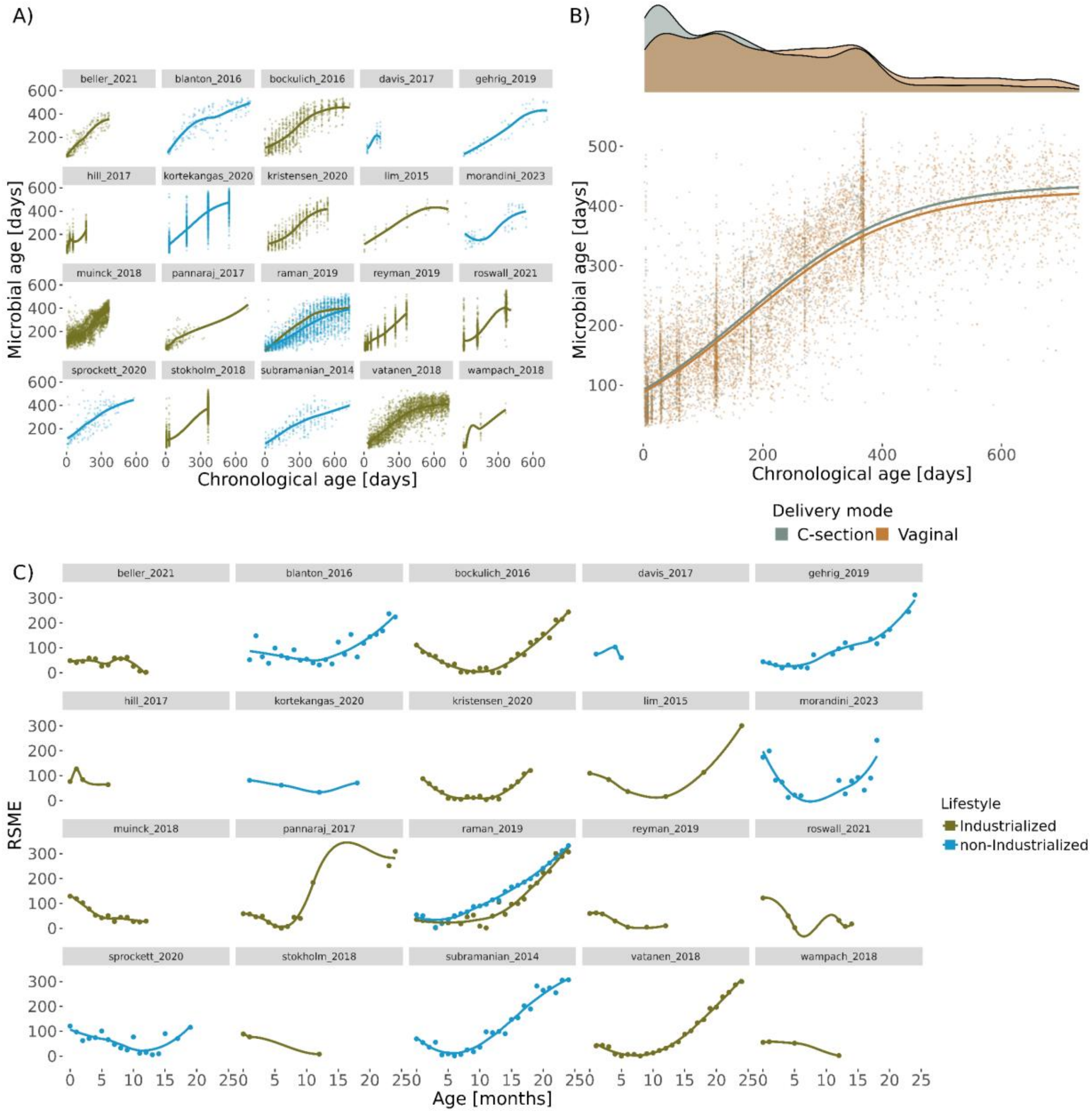

Supplementary Figure S3. Biological and technical effects on microbial age prediction. **A)** Microbial age development over chronological age plotted per study. Locally estimated scatterplot smoothing (LOESS) curves are fit to visualize temporal trends. **B)** Microbial age development over chronological age for industrialized individuals with available information about delivery mode. A logistic growth curve was fit to visualize the nonlinear trajectory of the maturation dynamic over chronological age. **C)** RMSE of microbial age prediction along chronological age in monthly bins per study. Locally estimated scatterplot smoothing (LOESS) curves are fit to visualize temporal trends. RMSE, unlike R^2, shows an age-dependent bias towards the mean, especially for values at the upper and lower end of the range.

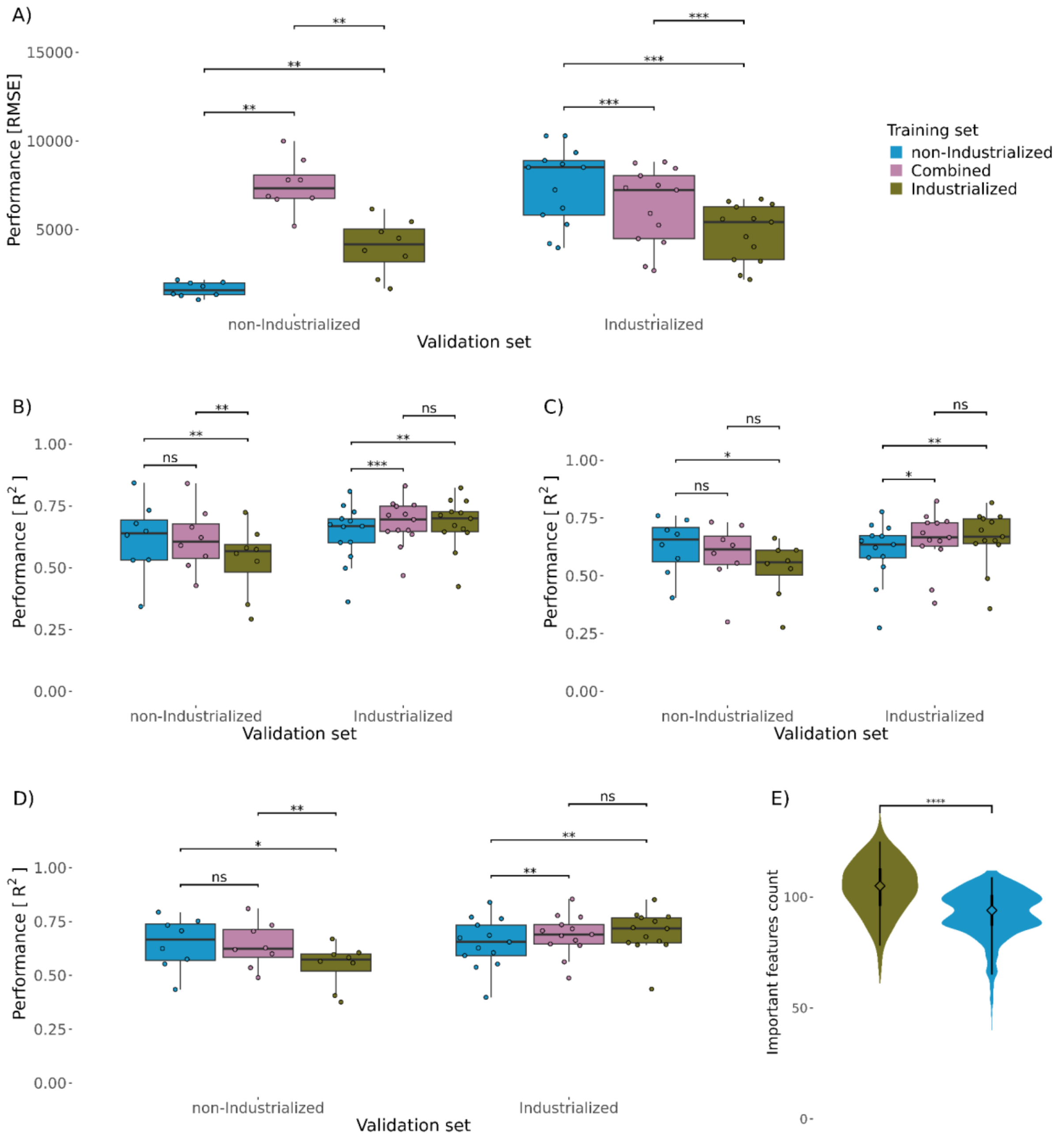

Supplementary Figure S4. Results for downsampling both lifestyle groups to the same number of studies, subjects and samples. **A)** Performance of microbial age modelling with lifestyle specific models. Separate models were trained either on microbiomes from industrialized samples, non-industrialized samples, or a combined dataset. Performance was assessed using LODO-CV, where each study served as a validation set once for each model. Root mean squared error (RMSE) between microbial age versus chronological age was used as performance metrics and was calculated for each combination of model and validation set separately. Differences in model performances were tested with a paired Wilcoxon test and adjusted for multiple comparisons using Bonferroni correction (**padj*<0.1, ***padj*<0.05, ****padj*<0.01). **B)** Performance of microbial age modelling with lifestyle specific models on relative abundances on family level. Coefficient of determination (R^2) from a linear model of

rank transformed microbial age versus chronological age is used as performance metrics and was calculated for each combination of model and validation set separately. **C)** Performance of microbial age modelling with lifestyle specific models on relative abundances of read counts that were rarefied to 2,000 reads per sample. **D)** Performance of microbial age modelling with lifestyle specific models. Separate models were trained either on microbiomes from industrialized samples, non-industrialized samples, or a combined dataset. Each point represents the mean out of 50 downsampling iterations. **E)** Difference in the number of important features between industrialized and non-industrialized models trained on downsampled data. Each value represents one of 1.000 downsampling iterations. Significance was tested with a paired Wilcoxon test (*$p<0.1$, **$p<0.05$, ***$p<0.01$, ****$p<0.001$).

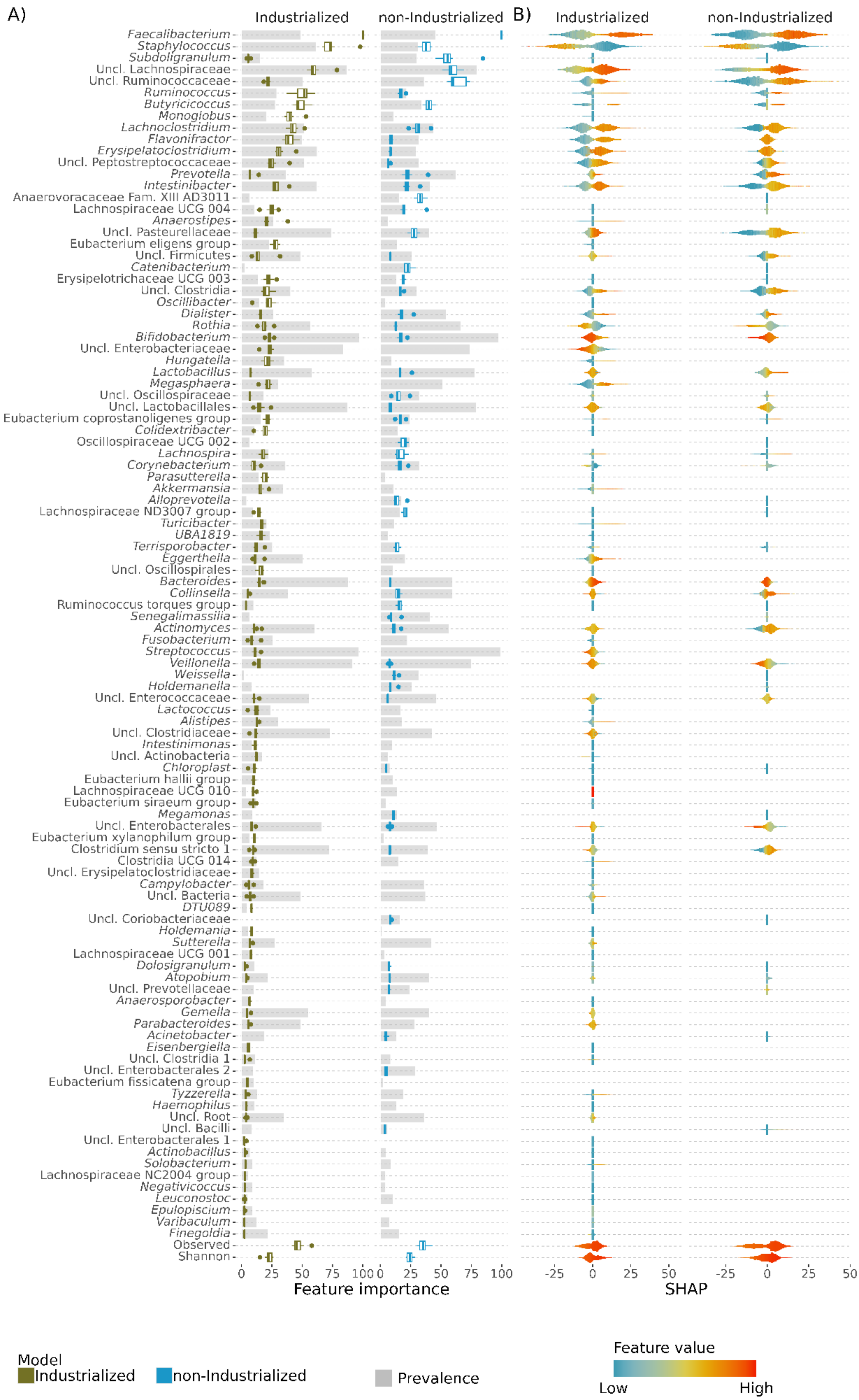
A)
Industrialized
non-Industrialized
B)
Industrialized
non-Industrialized
Faecalibacterium
Staphylococcus
Subdoligranulum
Uncl. Lachnospiraceae
Uncl. Ruminococcaceae
Ruminococcus
Butyricicoccus
Monoglobus
Lachnoclostridium
Flavonifractor
Erysipelatoclostridium
Uncl. Peptostreptococcaceae
Prevotella
Intestinibacter
Anaerovoracaceae Fam. XIII AD3011
Lachnospiraceae UCG 004
Anaerostipes
Uncl. Pasteurellaceae
Eubacterium eligens group
Uncl. Firmicutes
Catenibacterium
Erysipelotrichaceae UCG 003
Uncl. Clostridia
Oscillibacter
Dialister
Rothia
Bifidobacterium
Uncl. Enterobacteriaceae
Hungatella
Lactobacillus
Megasphaera
Uncl. Oscillospiraceae
Uncl. Lactobacillales
Eubacterium coprostanoligenes group
Colidextribacter
Oscillospiraceae UCG 002
Lachnospira
Corynebacterium
Parasutterella
Akkermansia
Alloprevotella
Lachnospiraceae ND3007 group
Turicibacter
UBA1819
Terrisporobacter
Eggerthella
Uncl. Oscillospirales
Bacteroides
Collinsella
Ruminococcus torques group
Senegalimassilia
Actinomyces
Fusobacterium
Streptococcus
Veillonella
Weissella
Holdemanella
Uncl. Enterococcaceae
Lactococcus
Alistipes
Uncl. Clostridiaceae
Intestinimonas
Uncl. Actinobacteria
Chloroplast
Eubacterium hallii group
Lachnospiraceae UCG 010
Eubacterium siraeum group
Megamonas
Uncl. Enterobacterales
Eubacterium xylanophilum group
Clostridium sensu stricto 1
Clostridia UCG 014
Uncl. Erysipelatoclostridiaceae
Campylobacter
Uncl. Bacteria
DTU089
Uncl. Coriobacteriaceae
Holdemania
Sutterella
Lachnospiraceae UCG 001
Dolosigranulum
Atopobium
Uncl. Prevotellaceae
Anaerosporobacter
Gemella
Parabacteroides
Acinetobacter
Eisenbergiella
Uncl. Clostridia 1
Uncl. Enterobacterales 2
Eubacterium fissicatena group
Tyzzerella
Haemophilus
Uncl. Root
Uncl. Bacilli
Uncl. Enterobacterales 1
Actinobacillus
Solobacterium
Lachnospiraceae NC2004 group
Negativicoccus
Leuconostoc
Epulopiscium
Variibaculum
Finegoldia
Observed
Shannon
0 25 50 75 100 0 25 50 75 100
Feature importance
-25 0 25 50 -25 0 25 50
SHAP
Model
Industrialized
non-Industrialized
Prevalence
Feature value
Low
High

Supplementary Figure S5. Relevance of important taxa in lifestyle specific models. All taxa identified as significantly important by Boruta in at least two models of LODO-CV for the specific lifestyle are displayed. **A)** Relative feature importance in random forest models trained separately on samples from individuals with an industrialized or a non-industrialized lifestyle. Grey bars indicate the prevalence of a specific taxon in each of the lifestyles. Importances are displayed only for features significantly important for the respective lifestyle. **B)** Violin plot for Shapley additive explanation (SHAP) values. Color indicates relative taxon abundance or alpha diversity, scaled per feature. Only values for features significantly important for the respective lifestyle are shown.

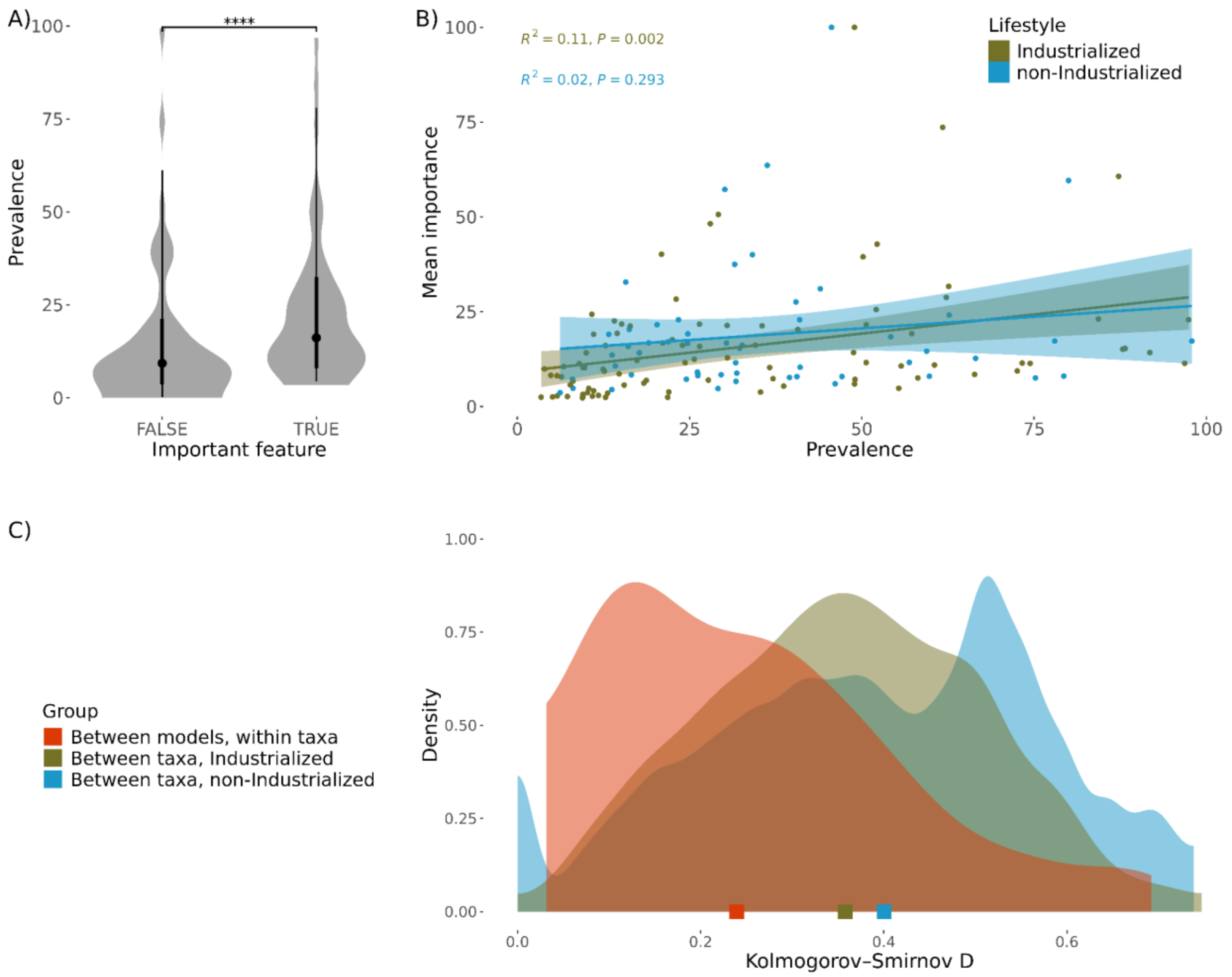

Supplementary Figure S6. A) Difference in prevalence of taxa important only in one lifestyle group. Prevalences are compared between the lifestyle where the feature is important and the lifestyle where it is not important. Diamonds represent medians, thick lines 50th percentiles, thin lines 95th percentiles. Significance was tested with a paired Wilcoxon test (**p*<0.1, ***p*<0.05, ****p*<0.01, *****p*<0.001). **B)** Correlation of mean relative feature importance and mean prevalence over all studies for all taxa from Figure S4. **C)** Density plots for the distributions of effect sizes derived from Kolmogorov-Smirnov tests comparing SHAP-value distributions. Comparisons were made either between models for the same taxa or between different taxa within individual models.

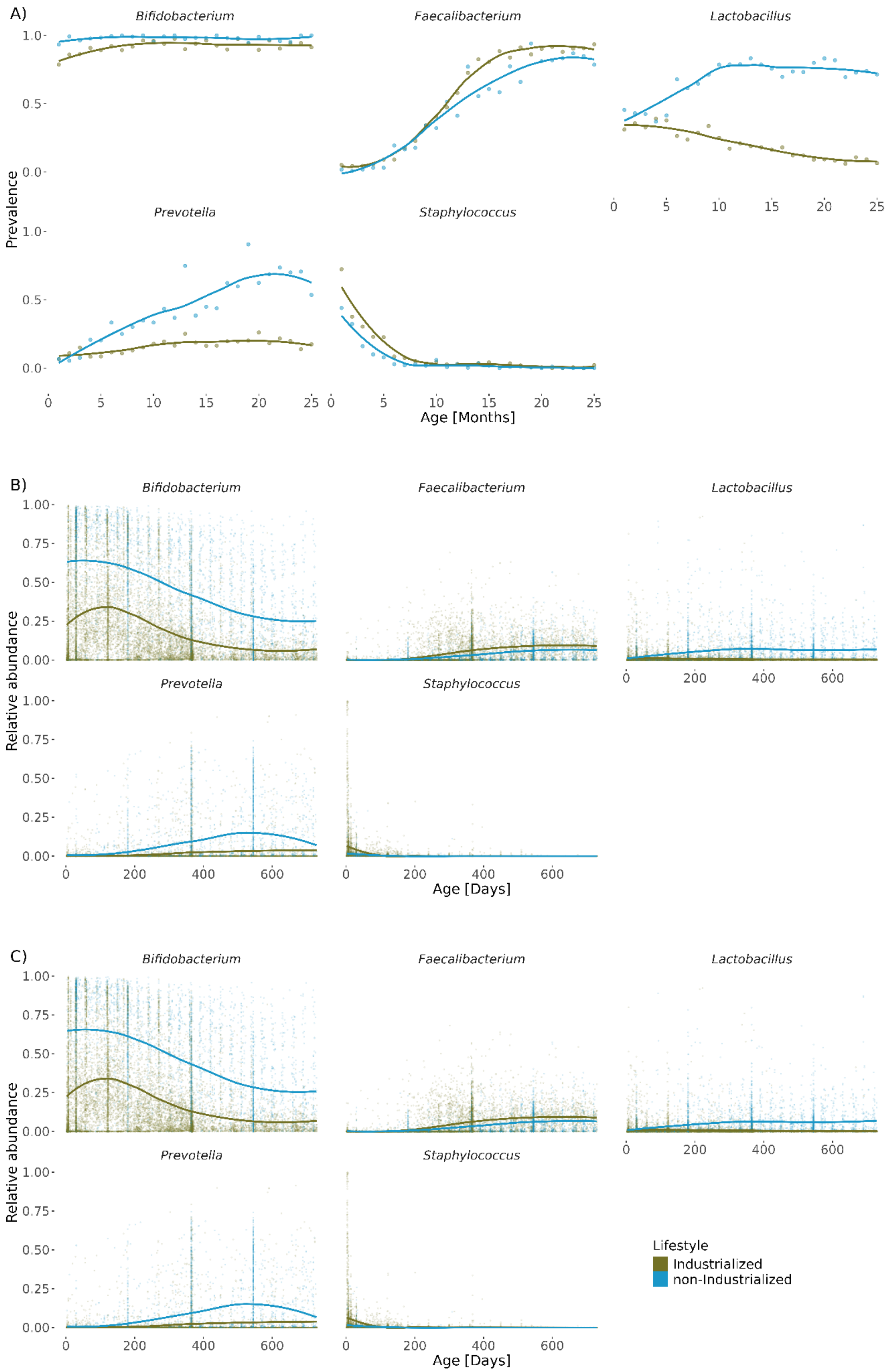
A)
Bifidobacterium
Faecalibacterium
Lactobacillus
Prevotella
Staphylococcus
Prevalence
Age [Months]
B)
Bifidobacterium
Faecalibacterium
Lactobacillus
Prevotella
Staphylococcus
Relative abundance
Age [Days]
C)
Bifidobacterium
Faecalibacterium
Lactobacillus
Prevotella
Staphylococcus
Relative abundance
Age [Days]
Lifestyle
Industrialized
non-Industrialized

Supplementary Figure S7. Longitudinal dynamics of taxa representative of the different colonization dynamics in samples from both lifestyles. **A)** Prevalence over chronological age binned in months in rarefied data. **B)** Relative abundance over chronological age in un-rarefied and **C)** rarefied data. LOESS curves are fit to visualize temporal trends.

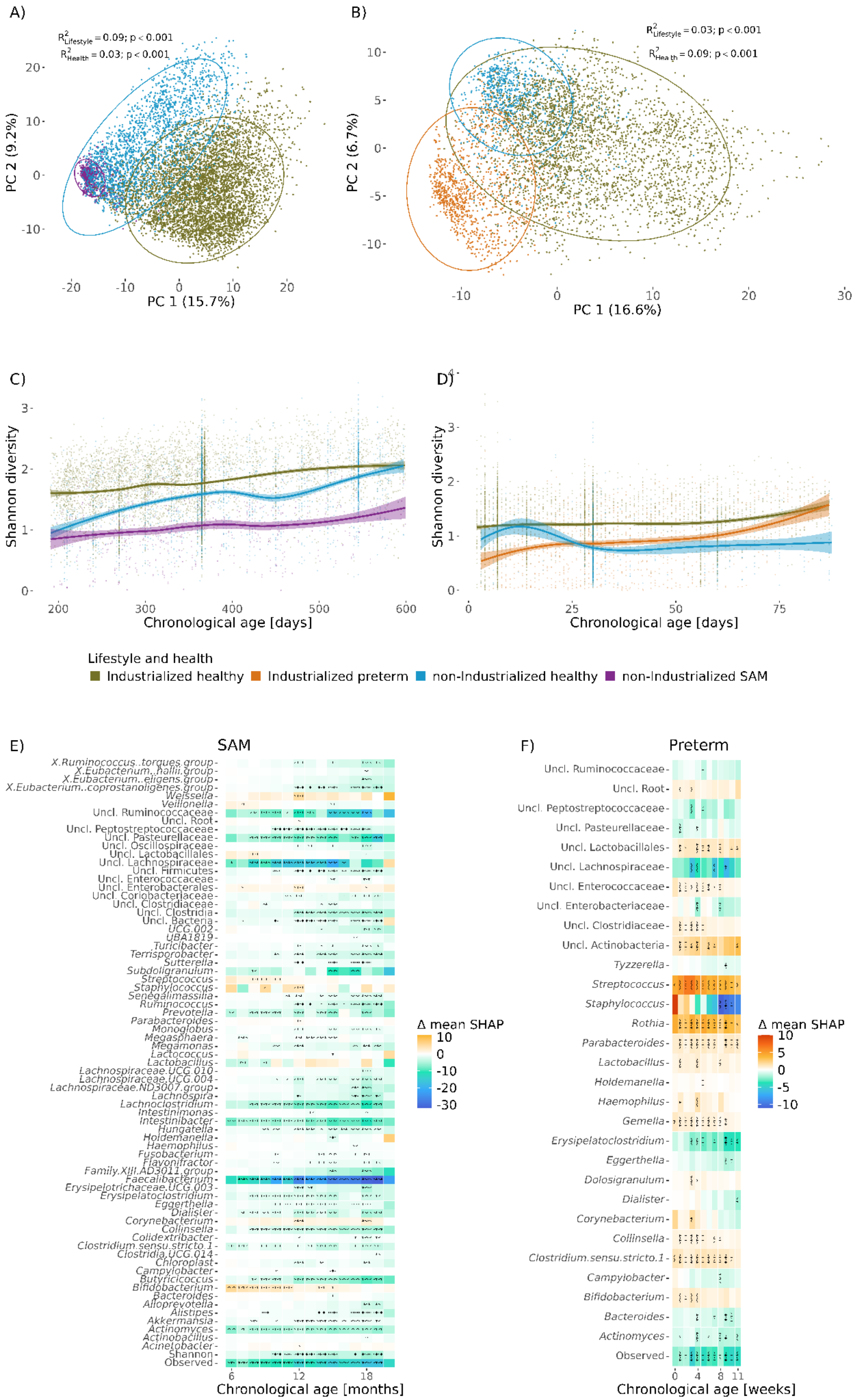

A)
$R^2_{Lifestyle}$ = 0.09; p < 0.001
R^2_{Health} = 0.03; p < 0.001
PC 2 (9.2%)
PC 1 (15.7%)
B)
$R^2_{Lifestyle}$ = 0.03; p < 0.001
R^2_{Health} = 0.09; p < 0.001
PC 2 (6.7%)
PC 1 (16.6%)
C)
Shannon diversity
Chronological age [days]
D)
Shannon diversity
Chronological age [days]
Lifestyle and health
Industrialized healthy
Industrialized preterm
non-Industrialized healthy
non-Industrialized SAM
E)
SAM
Δ mean SHAP
Chronological age [months]
F)
Preterm
Uncl. Ruminococcaceae
Uncl. Root
Uncl. Peptostreptococcaceae
Uncl. Pasteurellaceae
Uncl. Lactobacillales
Uncl. Lachnospiraceae
Uncl. Enterococcaceae
Uncl. Enterobacteriaceae
Uncl. Clostridiaceae
Uncl. Actinobacteria
Tyzzerella
Streptococcus
Staphylococcus
Rothia
Parabacteroides
Lactobacillus
Holdemanella
Haemophilus
Gemella
Erysipelatoclostridium
Eggerthella
Dolosigranulum
Dialister
Corynebacterium
Collinsella
Clostridium.sensu.stricto.1
Campylobacter
Bifidobacterium
Bacteroides
Actinomyces
Observed
Δ mean SHAP
Chronological age [weeks]

Supplementary Figure S8. Application of the microbial age model to health conditions. **A, B)** PCA on CLR-transformed raw counts colored by lifestyle and health condition for healthy infants and non-industrialized infants with SAM (A) and preterm industrialized infants (B). Healthy samples outside the age range of non-healthy samples were excluded. R^2 values indicate the proportion of variance explained in a Permutational multivariate analysis of variance (PERMANOVA) using a sequential model with the terms age, lifestyle, health and study. **C, D)** Shannon diversity calculated from rarefied counts aggregated to genus level, plotted over chronological age and stratified by lifestyle and health condition for healthy infants and non-industrialized infants with SAM (C) and preterm industrialized infants (D). Locally estimated scatterplot smoothing (LOESS) curves are fit to visualize temporal trends.**E)** Differences in mean SHAP-values between non-industrialized infants with SAM and healthy non-industrialized infants by month. Depicted are all taxa with a significant difference in at least one month. SHAP-values are based on the non-industrialized model. Differences in SHAP-values were tested for each age bin with a Wilcoxon test and adjusted for multiple comparisons using Bonferroni correction (**padj*<0.1, ***padj*<0.05, ****padj*<0.01, *****padj*<0.001). **F)** Differences in mean SHAP-values between preterm and full-term industrialized infants binned by week. Depicted are all taxa with a significant difference in at least one week. SHAP-values are based on the industrialized model.

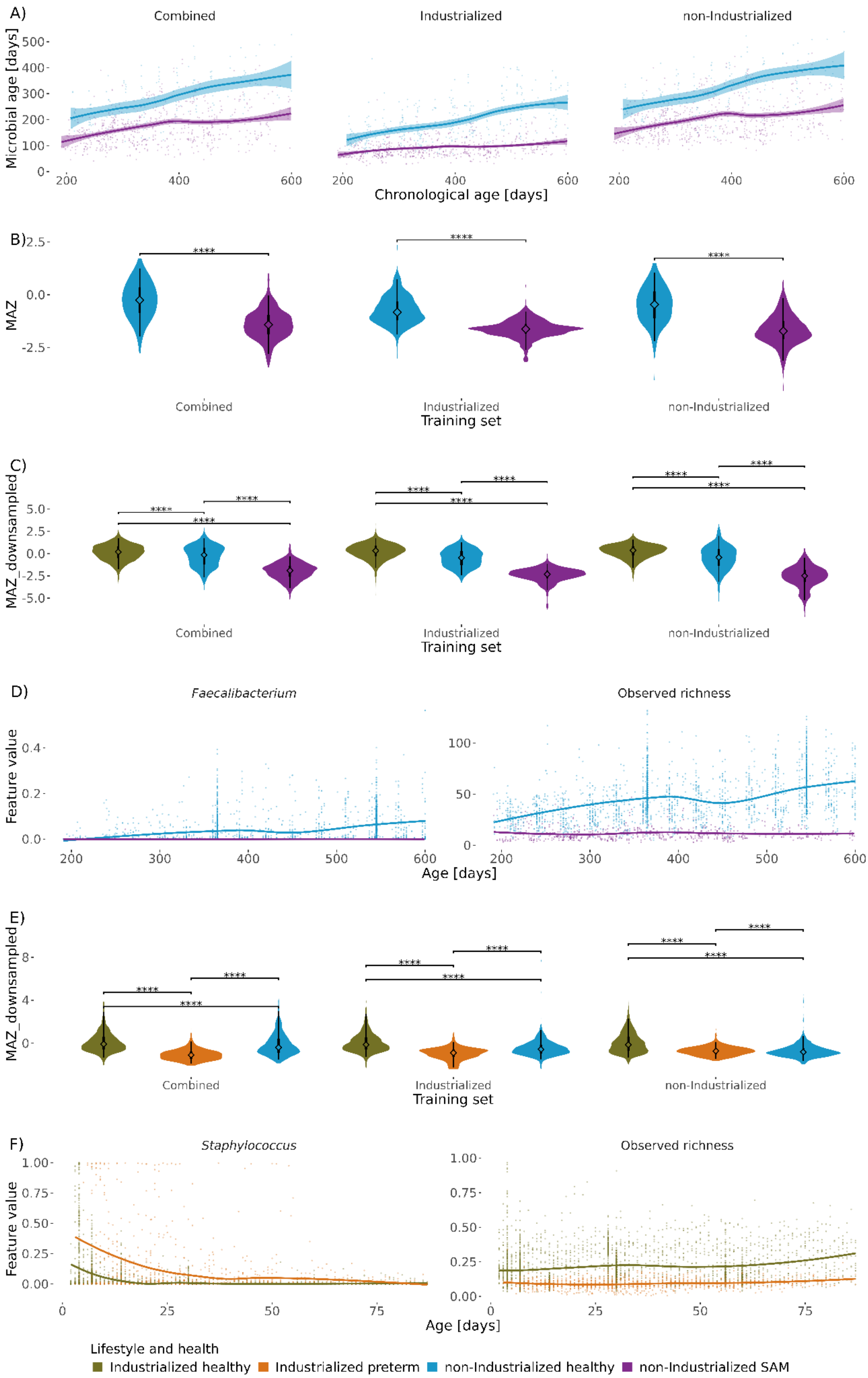

A)
Combined
Industrialized
non-Industrialized
Microbial age [days]
Chronological age [days]
B)
MAZ
Combined
Industrialized
non-Industrialized
Training set
C)
MAZ_downsampled
Combined
Industrialized
non-Industrialized
Training set
D)
Faecalibacterium
Observed richness
Feature value
Age [days]
E)
MAZ_downsampled
Combined
Industrialized
non-Industrialized
Training set
F)
Staphylococcus
Observed richness
Feature value
Age [days]
Lifestyle and health
Industrialized healthy
Industrialized preterm
non-Industrialized healthy
non-Industrialized SAM

Supplementary Figure S9. A) Predicted age for healthy and SAM infants in studies containing both healthy and SAM infants. **B)** Differences in MAZ between healthy and malnourished infants in studies containing both healthy and SAM infants. Diamonds represent medians, thick lines 50th percentiles, thin lines 95th percentiles. Significance was tested with a paired Wilcoxon test and adjusted for multiple comparisons using Bonferroni correction (**padj*<0.1, ***padj*<0.05, ****padj*<0.01, *****padj*<0.001). **C)** Differences in MAZ between SAM and healthy infants from both lifestyles predicted with a combined and the lifestyle specific models. MAZ was calculated based on median and standard deviation calculated from 50 randomly selected healthy samples per bin. **D)** Relative abundance over time for selected taxa and observed richness driving a delay in maturation in SAM infants. **E)** Differences in MAZ between preterm and healthy infants from both lifestyles predicted with a combined and the lifestyle specific models. MAZ was calculated based on median and standard deviation calculated from 50 randomly selected healthy samples per bin. **F)** Relative abundance over time for selected taxa and observed richness driving a delay in maturation preterm infants plotted over chronological age. LOESS curves are fit to visualize temporal trends.

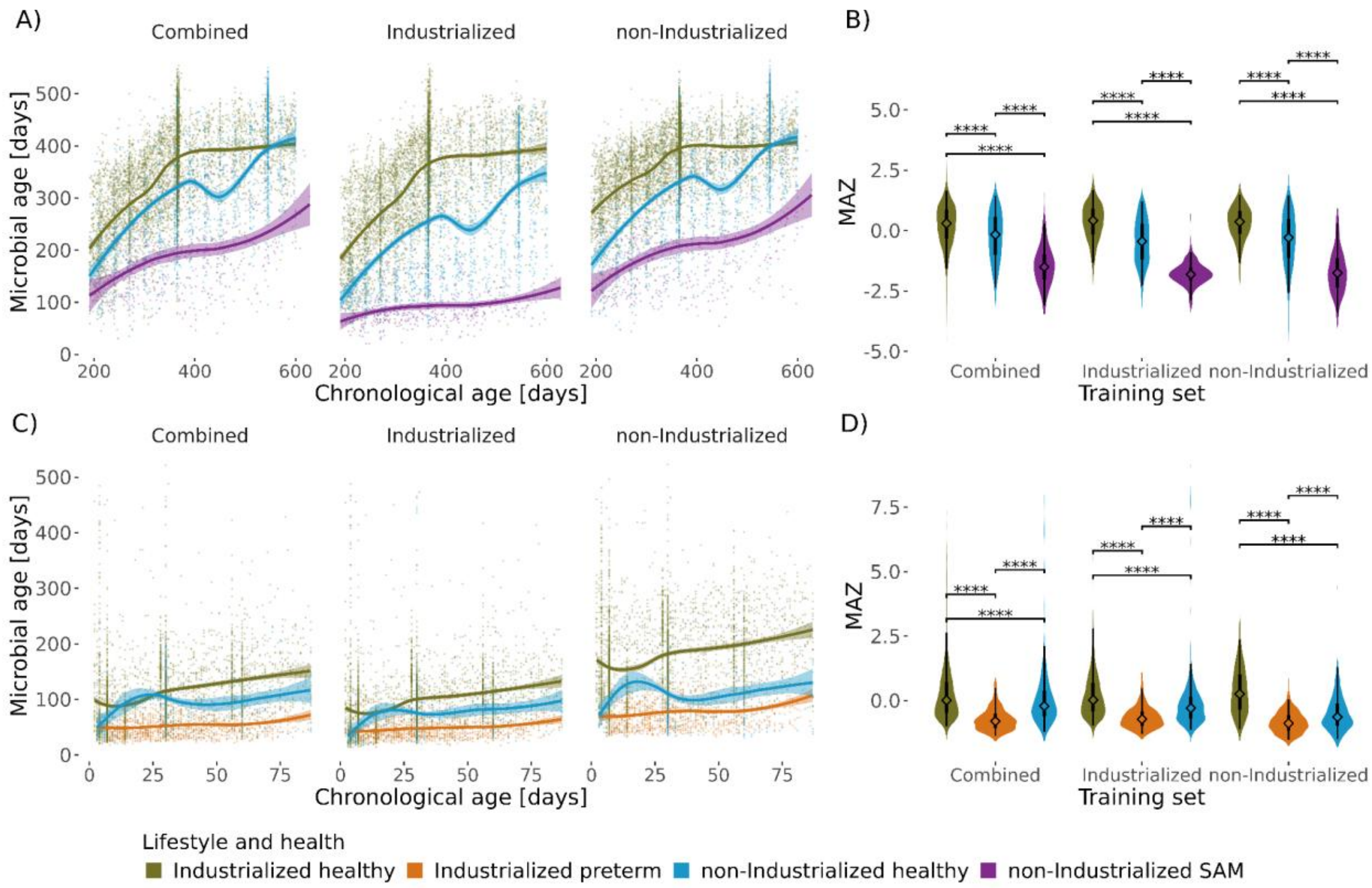

Supplementary Figure S10: A) Microbial age development over chronological age in combined and lifestyle specific models for healthy industrialized and non-industrialized infants and non-industrialized infants with acute severe malnutrition (SAM). Models were trained 50 times on downsampled versions of the training set where both lifestyles had a similar number of studies, individuals, and samples above and below one year of age. Each point represents the mean out of 50 downsampling iterations. LOESS curves are fit to indicate temporal trends. **B)** Differences in microbiome-for-age Z-scores (MAZ) between SAM and healthy infants from both lifestyles predicted with a combined and the lifestyle specific models trained on downsampled data. Diamonds represent medians, thick lines 50th percentiles, thin lines 95th percentiles. Differences in MAZ were tested with a Wilcoxon test and adjusted for multiple comparisons using Bonferroni correction (**padj*<0.1, ***padj*<0.05, ****padj*<0.01, *****p*<0.001).

C) Microbial age development over chronological age in combined and lifestyle specific models trained on downsampled data for full-term healthy industrialized and non-industrialized infants and preterm born industrialized infants. **D)** Differences in MAZ between preterm and healthy full-term infants from both lifestyles predicted with a combined and the lifestyle specific models trained on downsampled data.

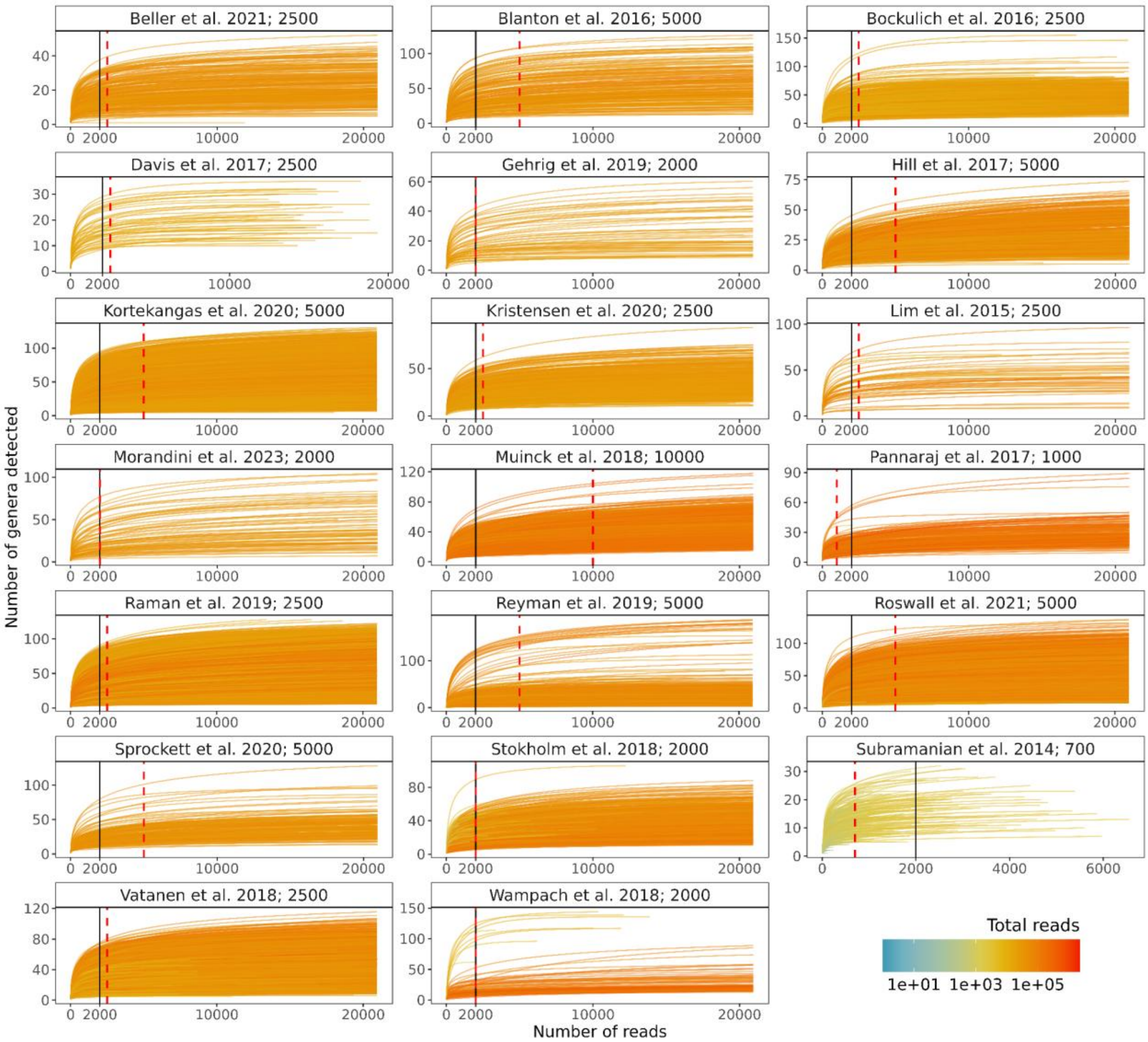

Supplementary Figure S11. Study specific rarefaction curves to determine study-specific filtering threshold. Filter thresholds are indicated by red dashed vertical lines and specified after the study name in the pane title. Gray solid line indicates the 2,000 reads threshold used for rarefaction. Color indicates the total number of taxonomically assigned reads for each sample.
